# Supplementary material for: Ru(II) Oxygen Sensors for Co(III) Complexes and Amphotericin B Antifungal Activity Detection by Phosphorescence Optical Respirometry
Source: Int J Mol Sci. 2023 May 14;24(10):8744. doi: 10.3390/ijms24108744 (PMC10218578; doi:10.3390/ijms24108744)
Supplement: Supplementary file 1 [file ijms-24-08744-s001.zip › ijms-2362536-supplementary.docx]

***Supplementary Materials***

Ru(II) Oxygen Sensors for Co(III) Complexes and
Amphotericin B Antifungal Activity Detection by Phosphorescence Optical Respirometry

Katarzyna Turecka^1*^, Agnieszka Chylewska ^2^, Aleksandra M. Dąbrowska ^2^, Rafał Hałasa^1^, Czesława Orlewska^3^ and Krzysztof Waleron^1^

^1^ Department of Pharmaceutical Microbiology, Faculty of Pharmacy, Medical University of Gdańsk, al. Hallera 107, 80-416 Gdańsk, Poland; [rafal.halasa@gumed.edu.pl](mailto:rafal.halasa@gumed.edu.pl), [krzysztof.waleron@gumed.edu.pl](mailto:krzysztof.waleron@gumed.edu.pl)

^2^ Department of Bioinorganic Chemistry, Faculty of Chemistry, University of Gdańsk, Wita Stwosza 63, 80-308 Gdańsk, Poland; [agnieszka.chylewska@ug.edu.pl](mailto:agnieszka.chylewska@ug.edu.pl), [aleksandra.dabrowska@ug.edu.pl](mailto:aleksandra.dabrowska@ug.edu.pl)

^3^ Department of Organic Chemistry, Faculty of Pharmacy, Medical University of Gdańsk, al. Hallera 107, 80-416 Gdańsk, Poland; [corl@gumed.edu.pl](mailto:corl@gumed.edu.pl)

***** Correspondence: [katarzyna.turecka@gumed.edu.pl](mailto:katarzyna.turecka@gumed.edu.pl); Tel.: (+48 58 349 19 73)

*
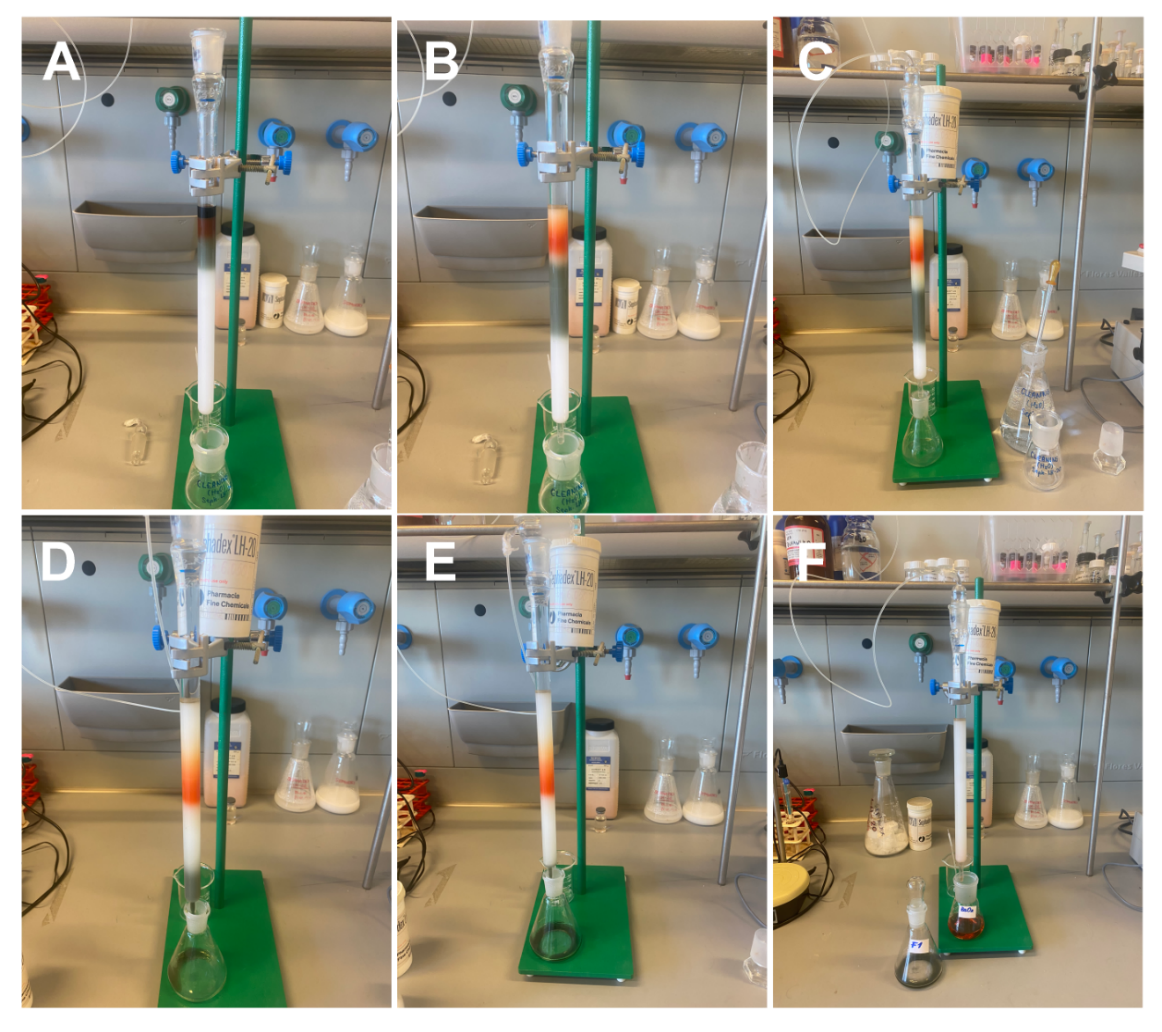
*

**Figure S1.** The {Ru^II^[DPP(SO_3_Na)_2_]_3_}Cl_2_ coordination compound separation process on a column with Sephadex LH-20; the water was used as an eluent; the green isomer reported in the literature as well as the expected BsOx complex product - the oxygen sensor – were obtained.

*
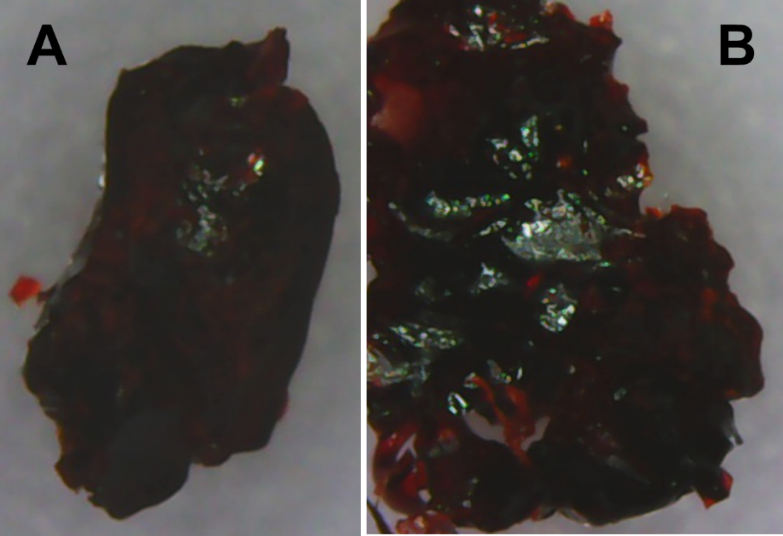
*

**Figure S2.** The microscopic pictures of the solid state of the oxygen biosensor resynthesized - {Ru[DPP(SO_3_Na)_2_]_3_(10H_2_O)}Cl_2·_12H_2_O: **A** and **B** present the different amorphous solid images.


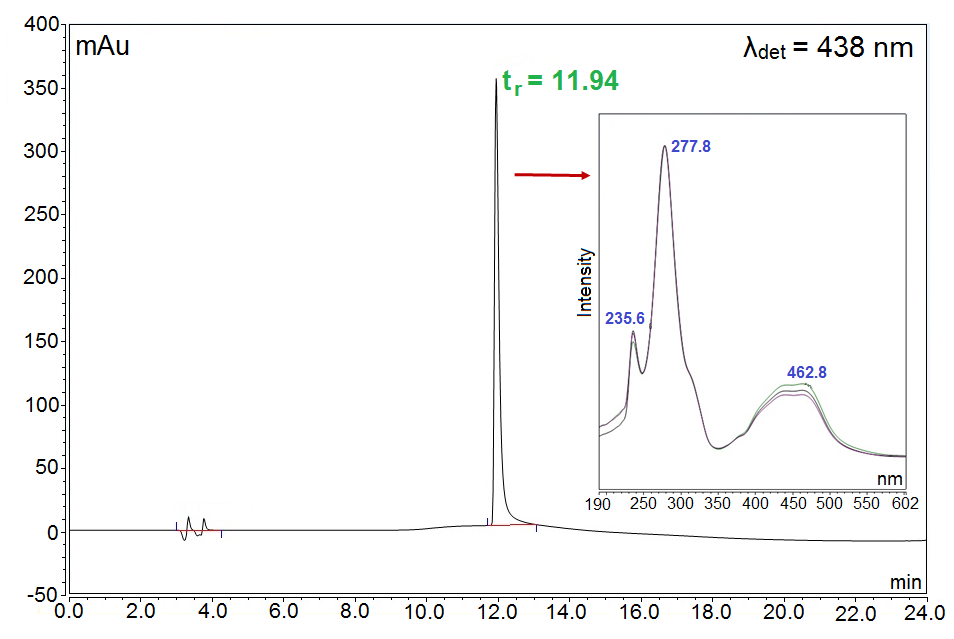


| **Retention time [min]** | **Peak Area [mAu·min]** | **Relative Area [%]** |
| --- | --- | --- |
| 3.30 | 0.1622 | 0.28 |
| 11.94 | 56.841 | 99.72 |

**Figure S3*.*** UHPLC chromatogram obtained (WVL-438 nm) for BsOx purity identification together with the BsOx UV-Vis spectra registered during this type of analysis.

**Table S1.** The data was obtained from UHPLC analyses.

**

**

**Figure S4.** The absorption spectrum of an aqueous solution of {Ru^II^[DPP(SO_3_Na)_2_]_3_}Cl_2_ studied.


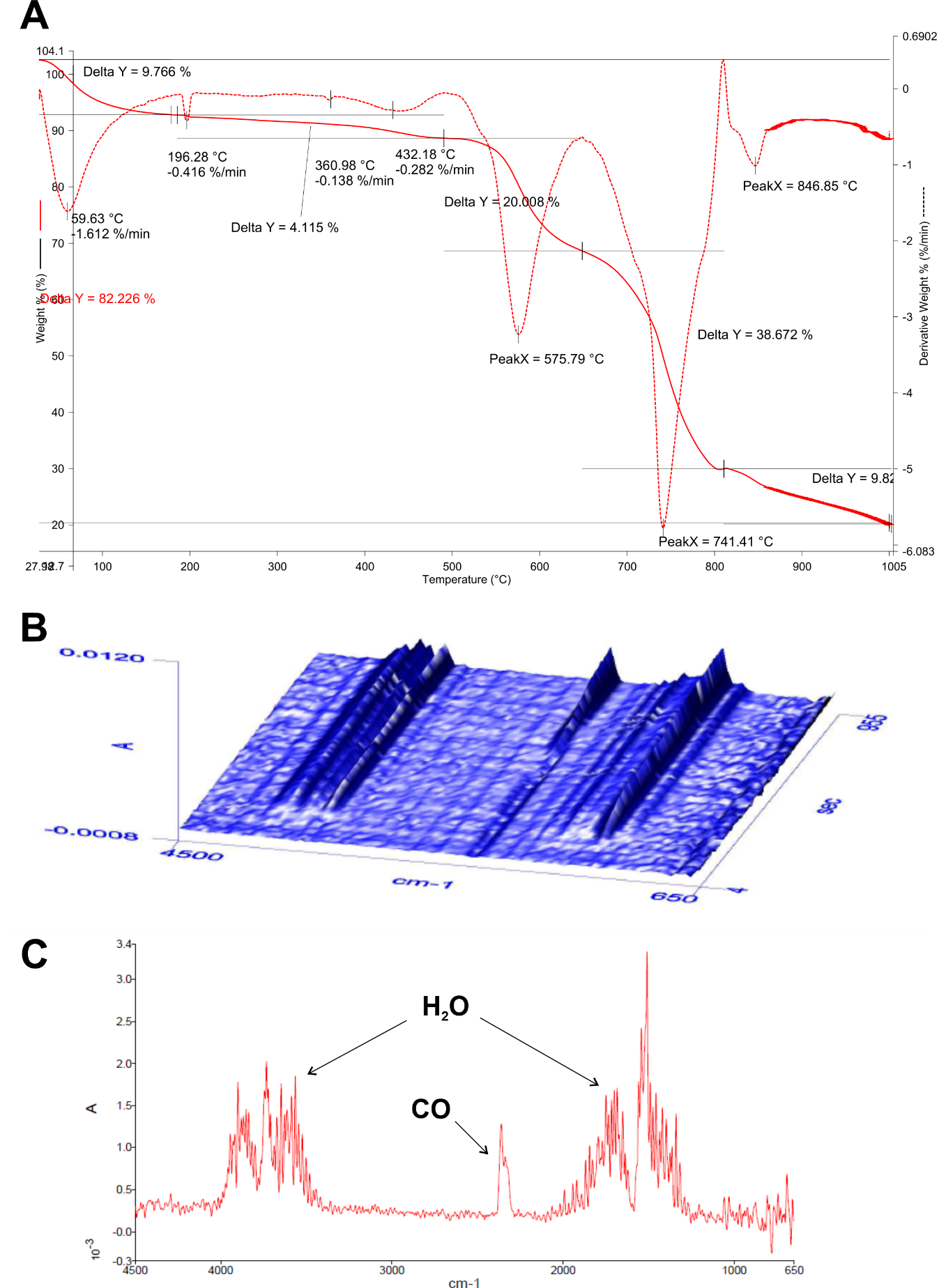


**Figure S5.** The hydration state establishing by using TG/IR measurements: **A.** the subsequent loss of BsOx complex sample weight registered; the first step of decomposition is the evidence of the BsOx hydrate form of solid received; **B.** the presentation of the 3D spatial IR spectra fragment of gaseous products generated by TG measurement; **C.** IR spectra of H_2_O and CO emitted from BsOx complex in 704.56 s of TG/IR analysis; the results presented in **B** and **C** were obtained for 1.607 mg of BsOx sample weight.





**Figure S6*.*** Voltamperometric curve of DMSO solution of the BsOx complex (10^-3^ M with addition of the standard electrolyte for the non-aqueous medium, 0.1 M TBAP) recorded by using glassy carbon working electrode (Ø = 2 mm GCE, scan rate: 100 mV/s).

***

***

**Figure S7.** ATR spectrum of ruthenium(II) complex synthesized. The BsOx solid sample was used to register the oscillatory vibration bands.


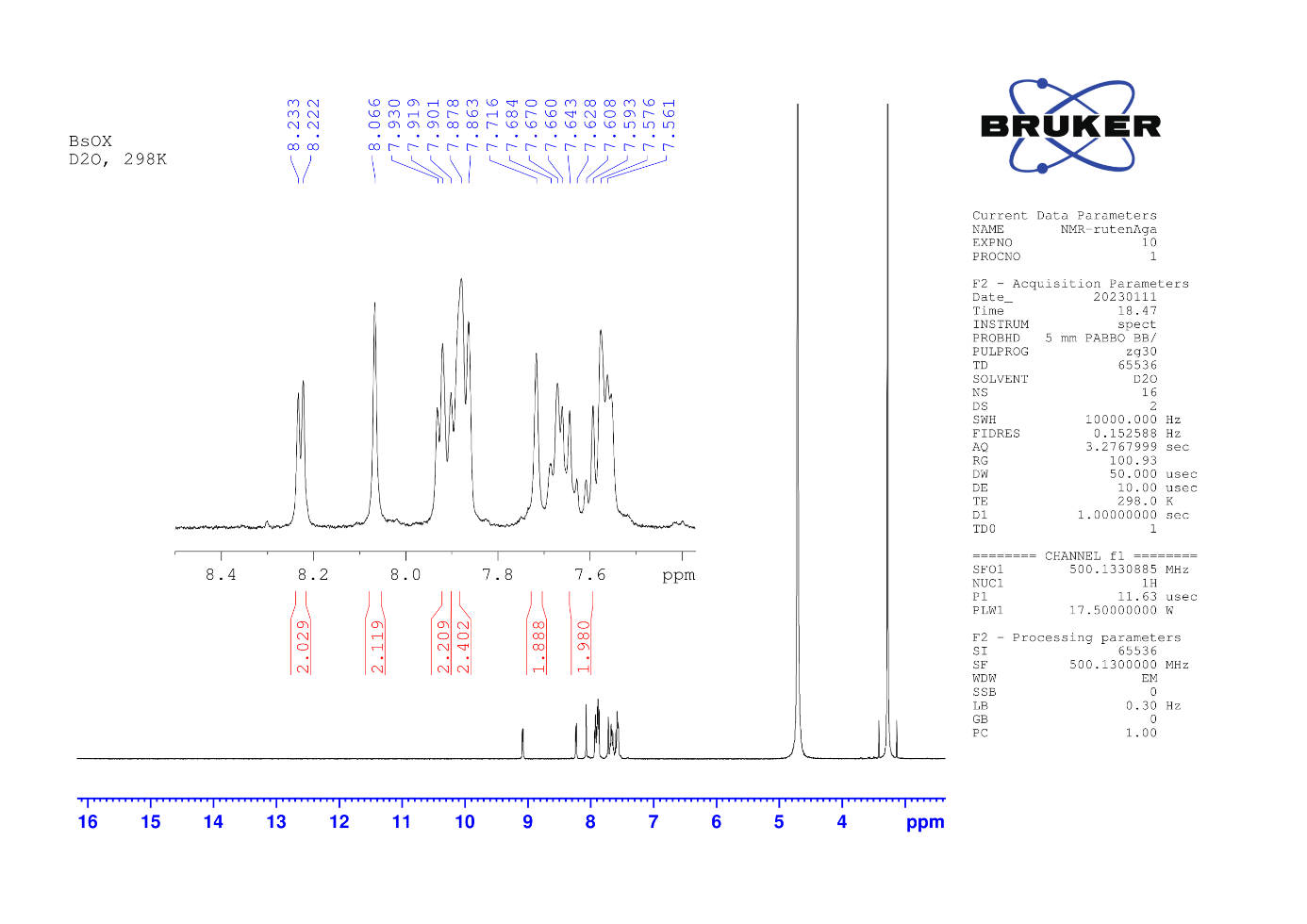


**Figure S8.** ^1^H NMR spectrum of BsOx with proton integration for {Ru[L]_1/3_}Cl_2_ (where L: C_24_H_14_N_2_Na_2_O_6_S_2_·3H_2_O) registered in D_2_O at 298K. The complete description was included in the manuscript’s main text (see the 2.2. section); the signals at 4.69 ppm for D_2_O as a solvent; 3.27 ppm for H_2_O hydrate as well as the typical H_2_O signal positions associated with D_2_O.

***
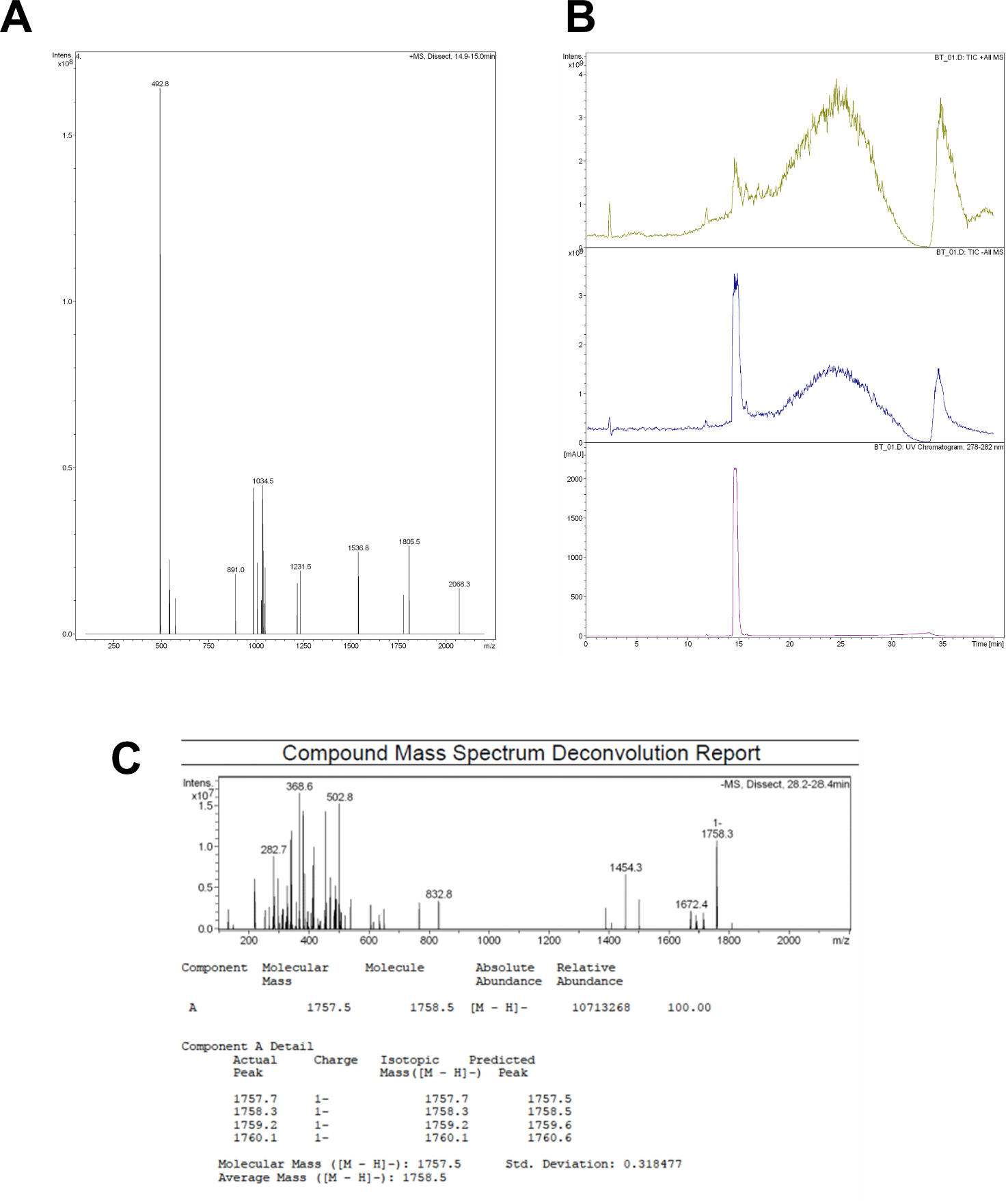
***

**Figure S9.** The LCMS results for BsOx registered after 15 minutes **A.** The m/z peaks were observed for multiple ionization of the compound. **B.** chromatograms: top – the water background; middle - compound together with the background; bottom – the aqueous solution signal for pure BsOx complex; the background is excluded (15 minutes); **C**. the selected MS negative mode mass spectrum of BsOx included in the deconvolution report for BsOx complex: *found:* 1758.5 [M-Na^+^]; *calc.* 1781.5 [M].

***
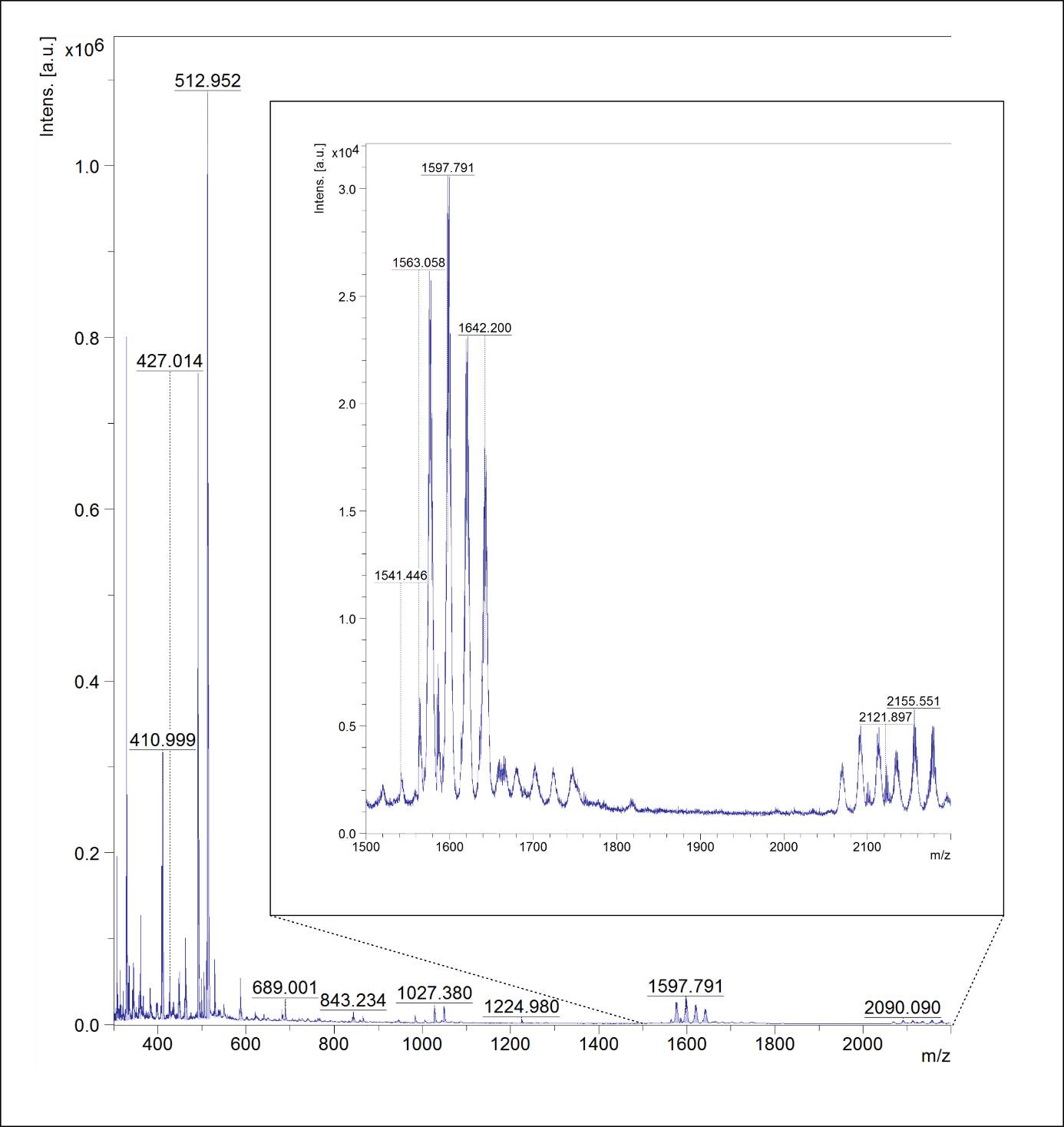
***

**Figure S10.** MALDI-TOF mass spectrum (DHB matrix) obtained for BsOx probe (multistep ionization process was confirmed by both results of analyses MALDI and QTOF, respectively).





**Figure S11.** ATR spectrum of ruthenium(II) and DPP complex synthesized. The Box solid sample was used to register the oscillatory vibration bands.





**Figure S12.** The absorption spectra of [Ru^II^(DPP)_3_]Cl_2_ studied (Box) aqueous solutions with different concentrations.


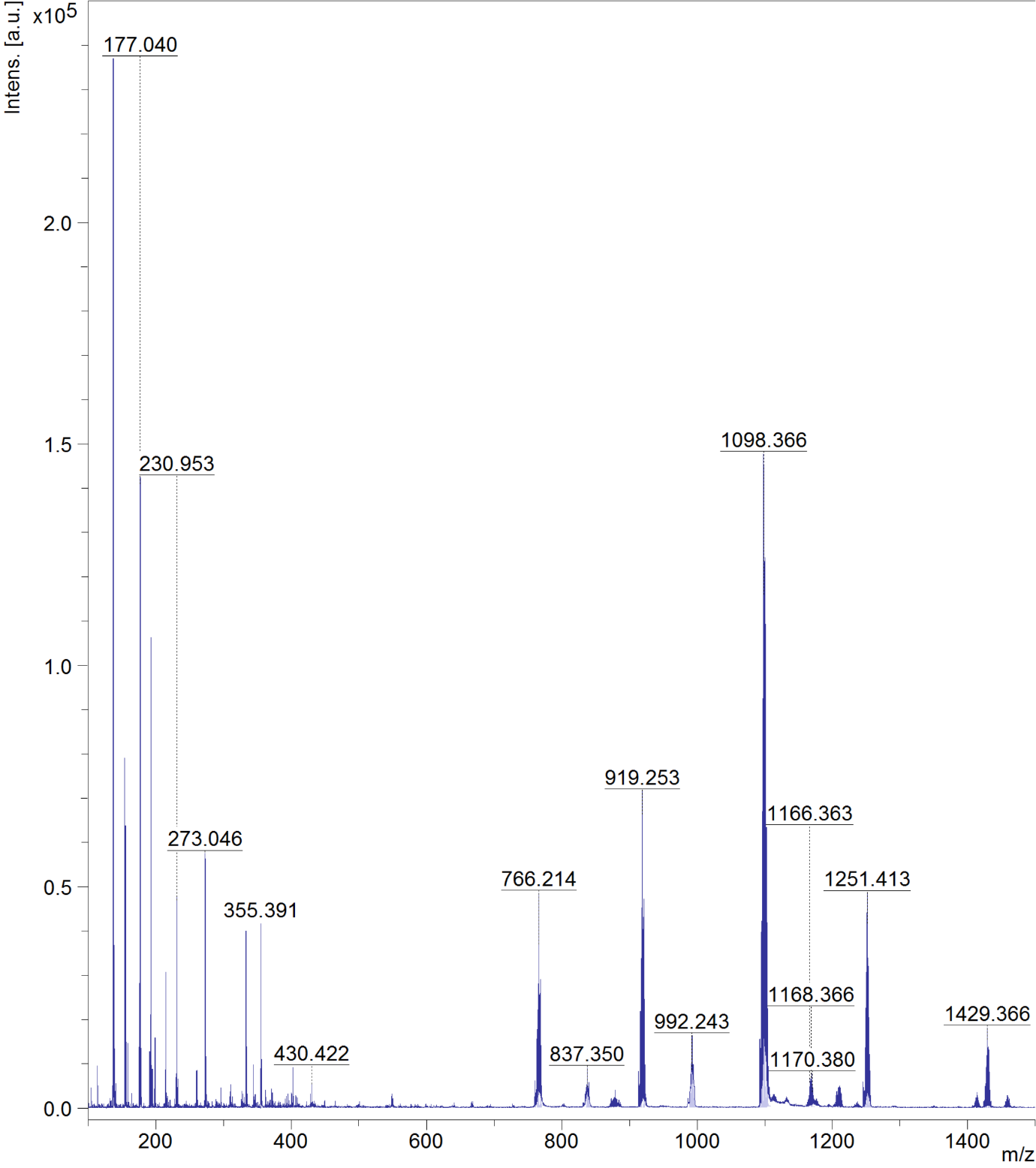


**Figure S13.** MALDI-TOF mass spectrum (DHB matrix) obtained for Box probe; m/z signals found (calc.): [M+H] 1170.38 (1170.24); [M-2Cl] 1098.37 (1098.24).

*

*

**Figure S14.** ATR spectrum of cobalt(III) complex synthesized with 1,3-diamine propane. The compound (**1**) solid sample was used to register the oscillatory vibration bands.


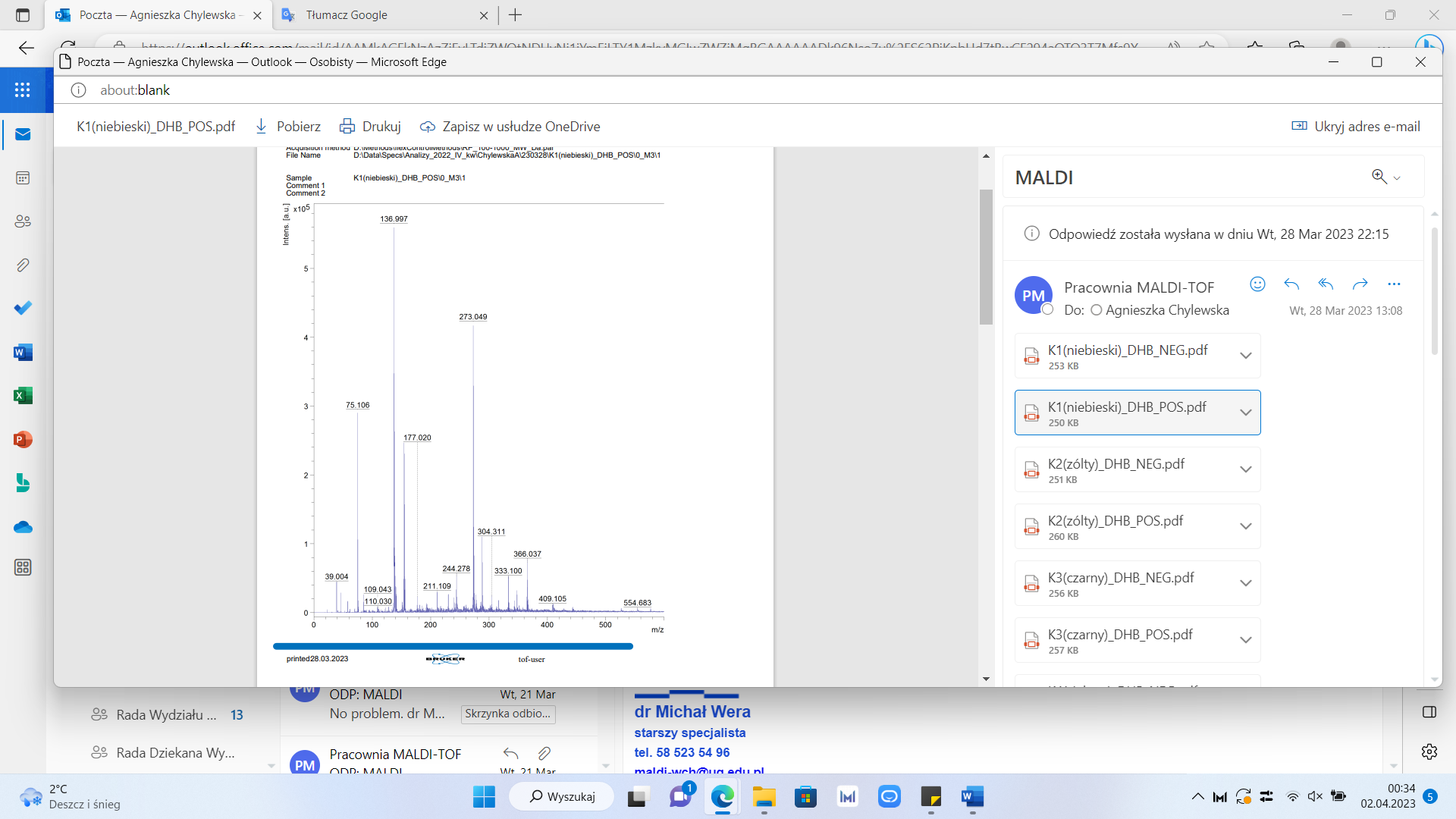


**Figure S15.** MALDI-TOF mass spectrum (DHB matrix) obtained for (**1**) Co(III) complex.





**Figure S16.** The absorption spectrum of an aqueous solution of coordination compound (**1**).

**
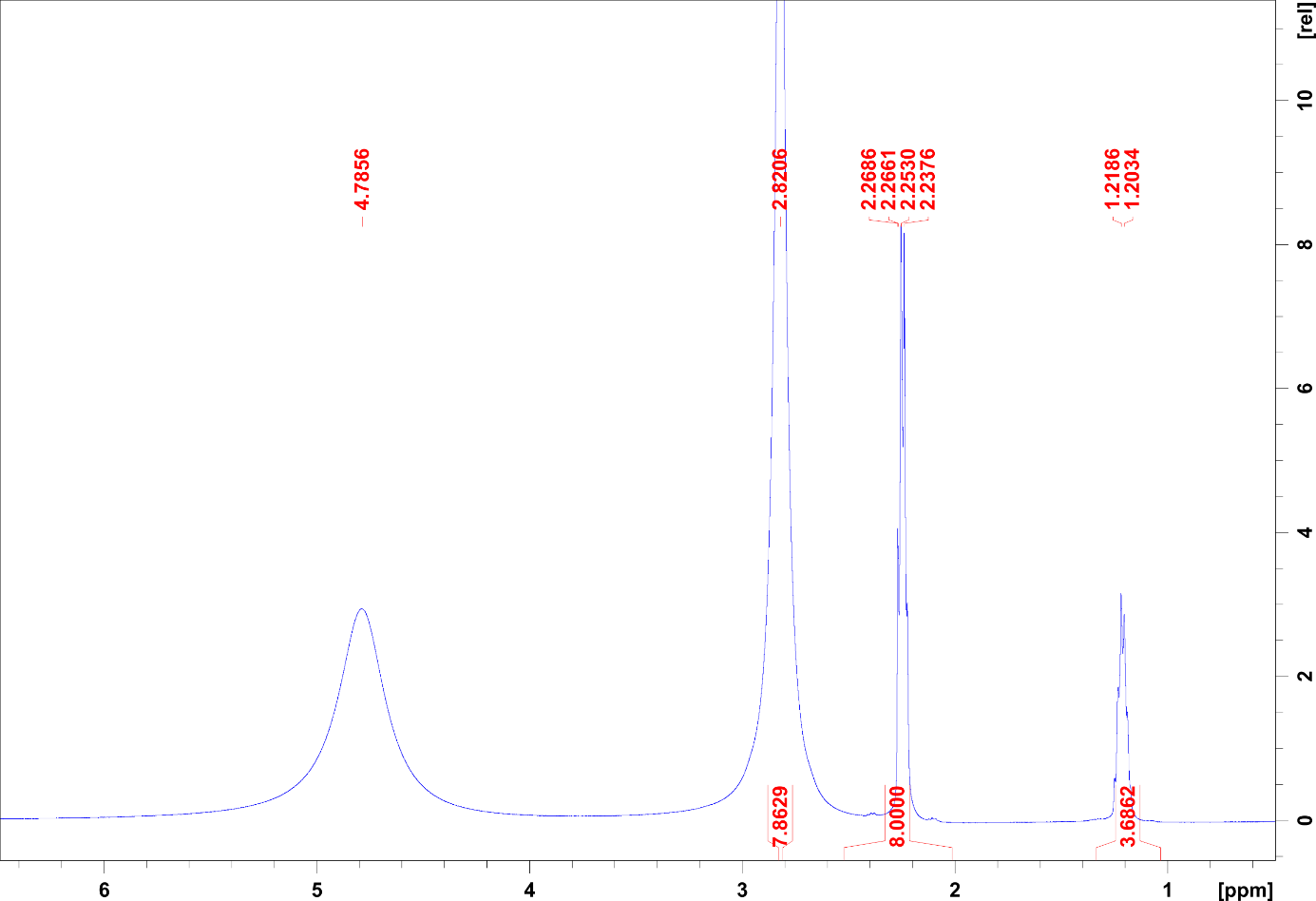
**

**Figure S17.** ^1^H NMR spectrum of compound (**1**) with proton integration registered in D_2_O at 298K.

**
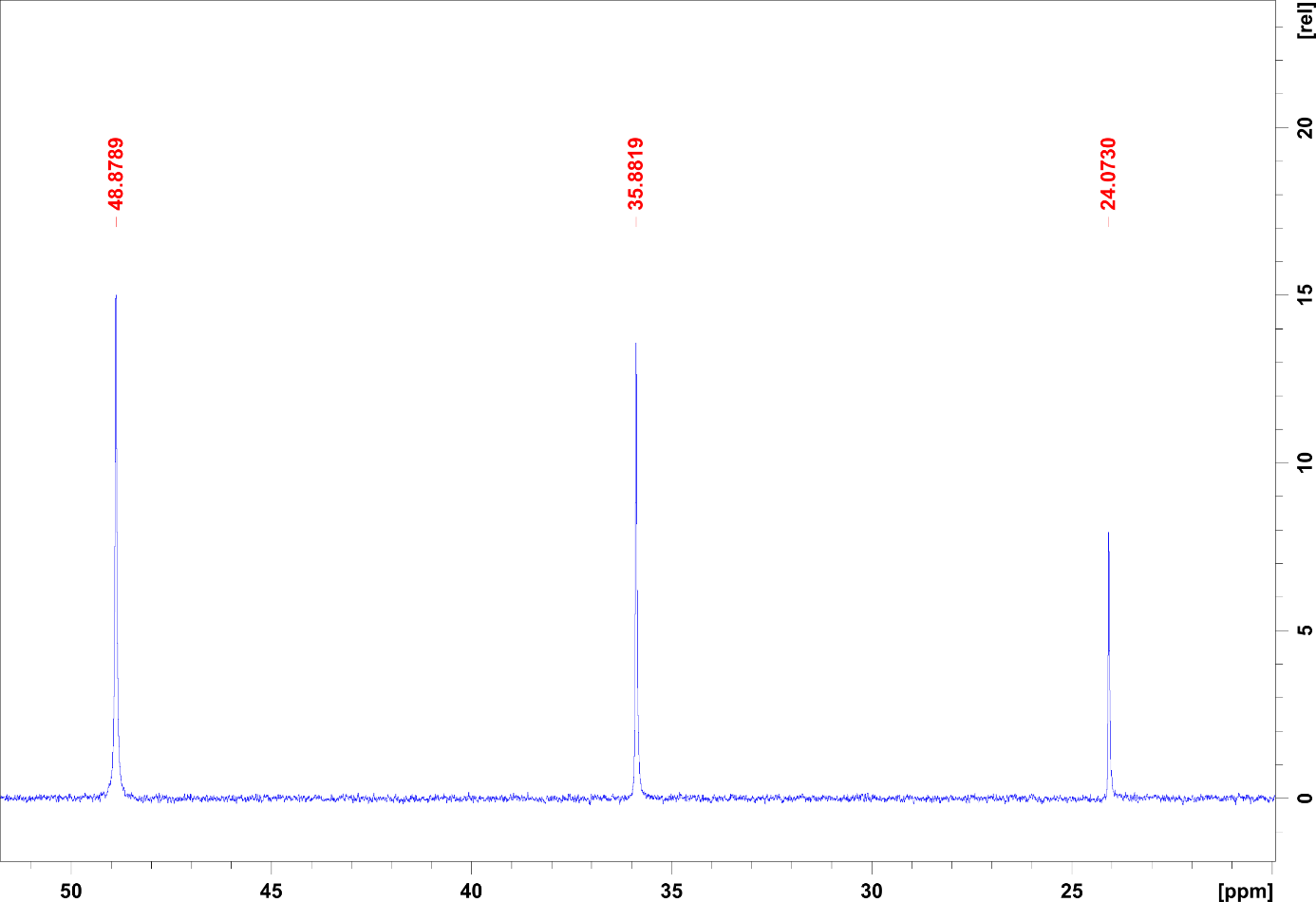
**

**Figure S18.** ^13^C NMR spectrum of compound (**1**) together with C-signals registered in D_2_O at 298K.

**
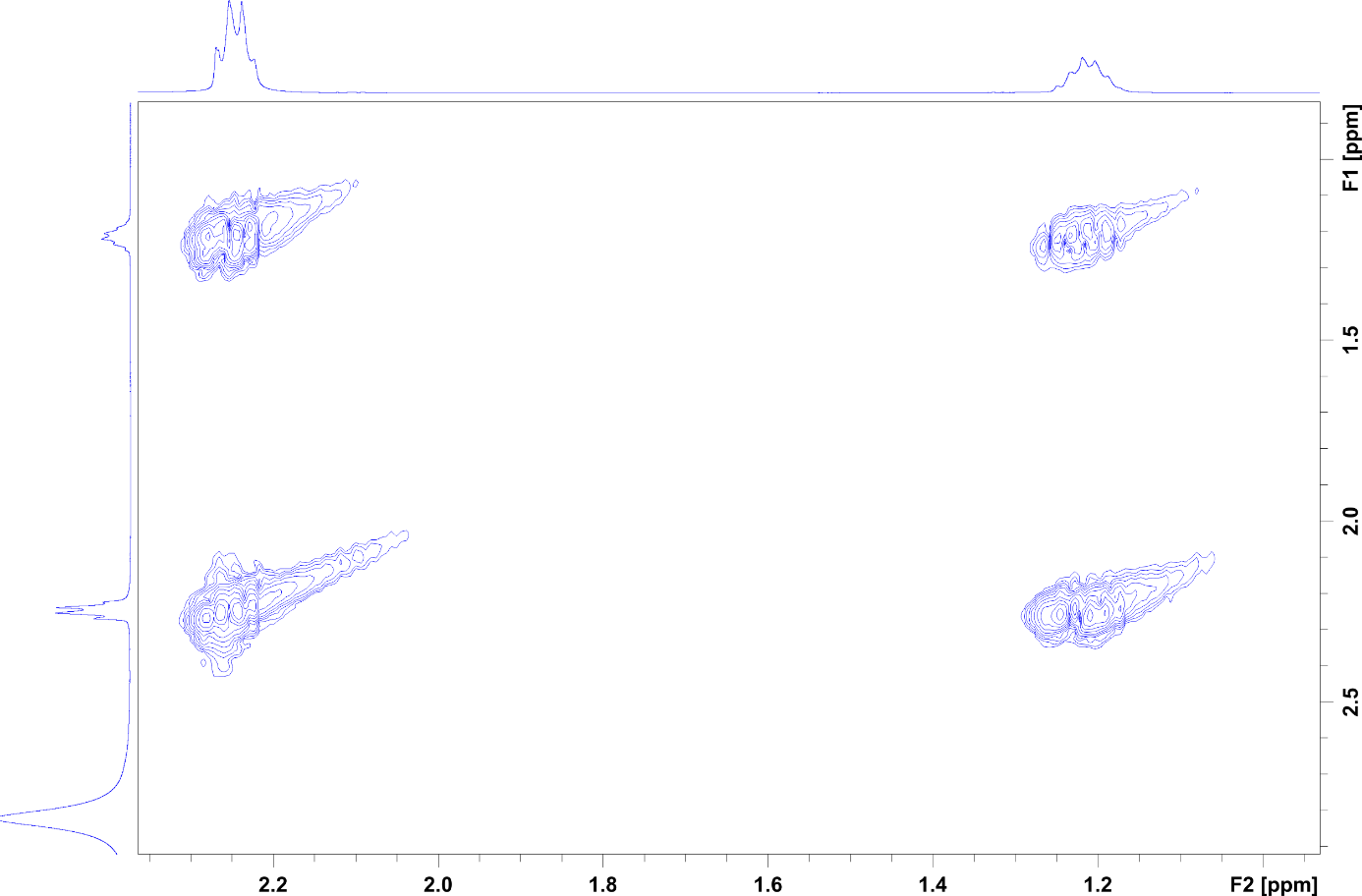
**

**Figure S19.** The 2D COSY spectrum of compound (**1**) registered in D_2_O at 298K.

**
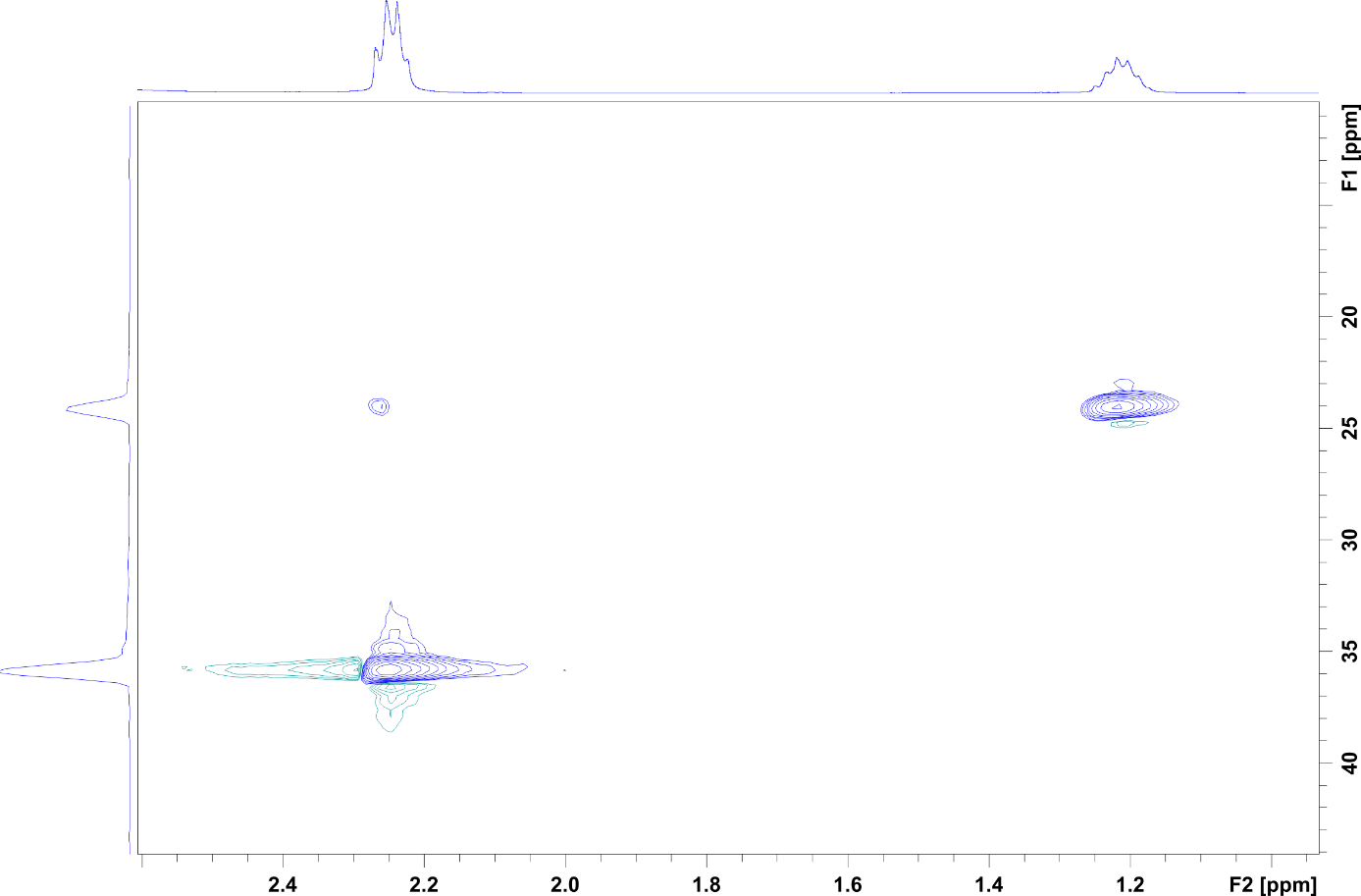
**

**Figure S20.** The main fragment of the HSQC spectrum of compound **(1)** indicated the C and H coupling only registered in D_2_O at 298K.





**Figure S21.** ATR spectrum of cobalt(III) complex synthesized with ethylenediamine. The compound (**2**) solid sample was used to register the oscillatory vibration bands.


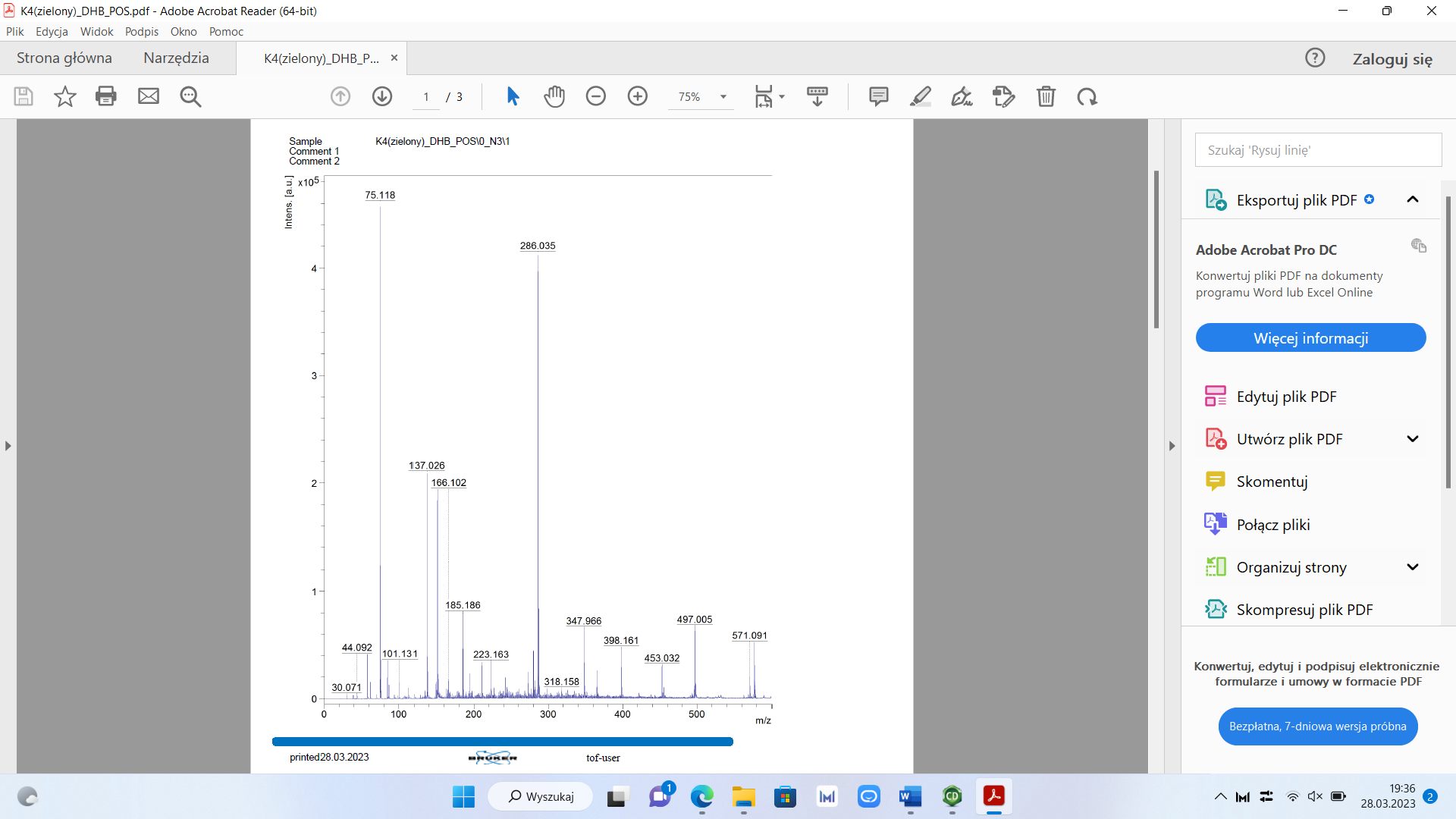


**Figure S22.** MALDI-TOF mass spectrum (DHB matrix) obtained for Co(III) complex (**2**).





**Figure S23.** The absorption spectrum of an aqueous solution of coordination compound (**2**).


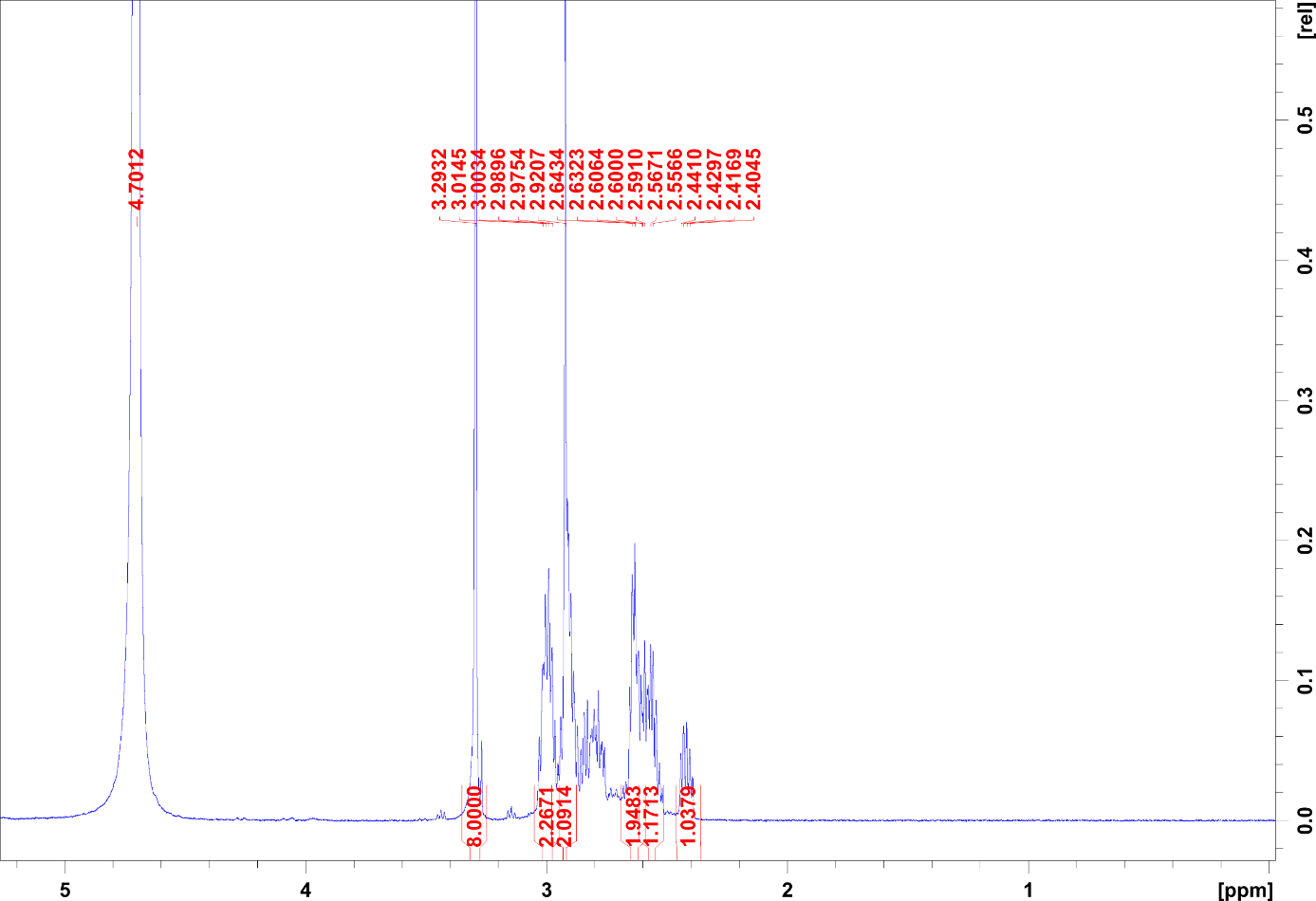


**Figure S24.** ^1^H NMR spectrum of compound (**2**) with proton integration registered in D_2_O at 298K.

**
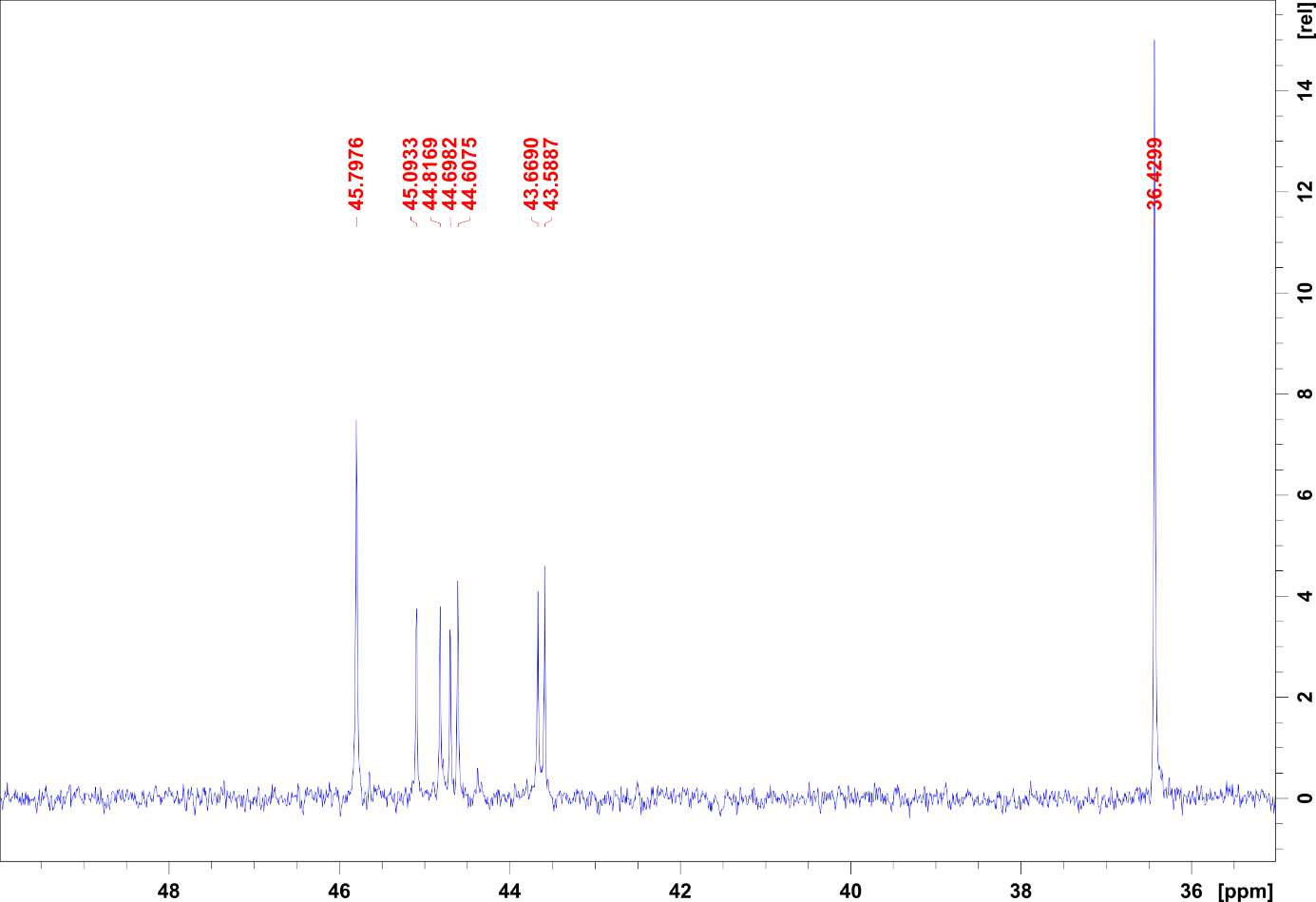
**

**Figure S25.** ^13^C NMR spectrum of compound (**2**) together with C-signals registered in D_2_O at 298K. The presence of two geometric isomers of (**2**) was identified in the solution (*cis* and *trans* form).

**
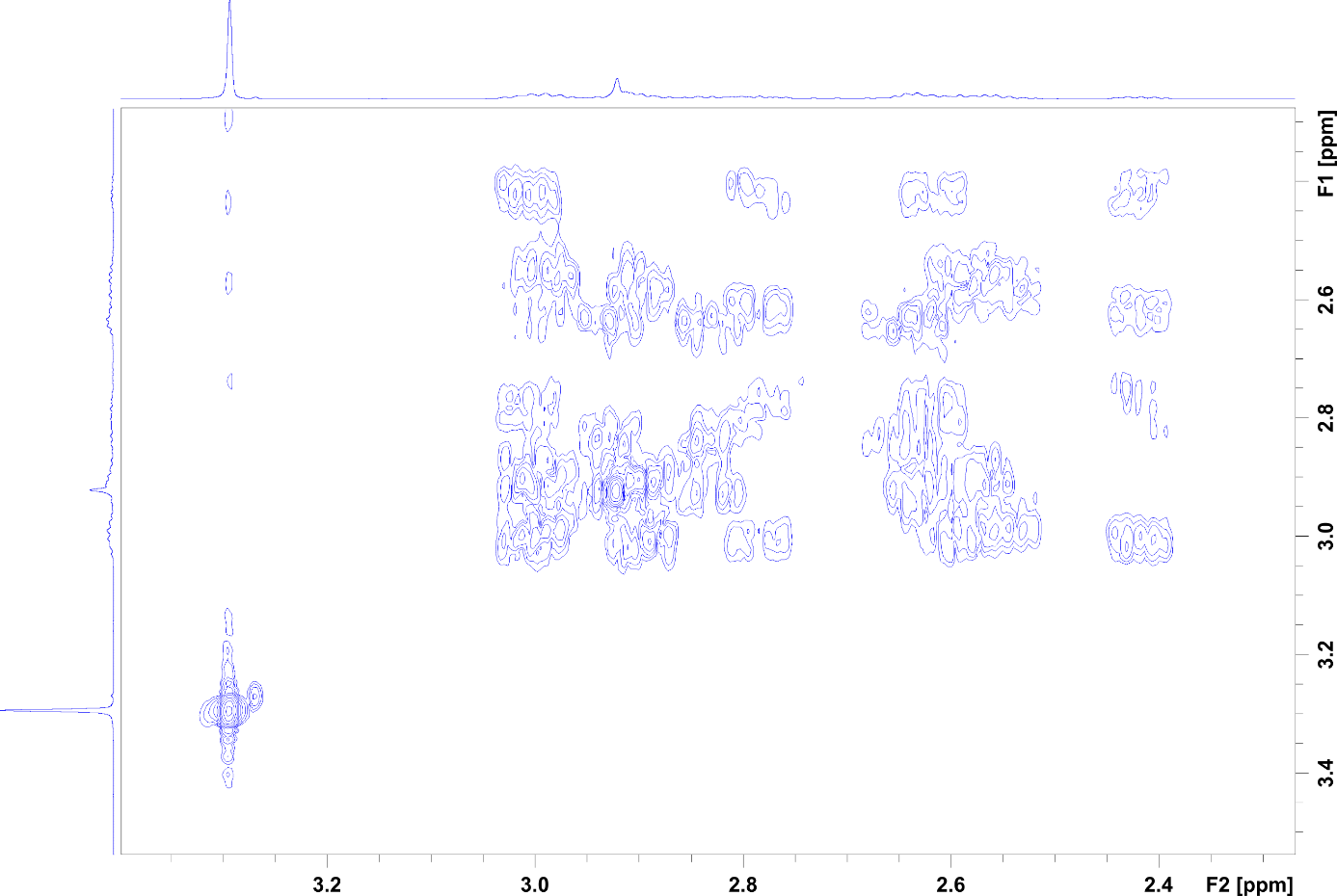
**

**Figure S26.** The 2D COSY spectrum of (**2**) is registered in D_2_O at 298K. Note that the NMR identified the *cis* and *trans* forms of compound (**2**) in the D_2_O.

**
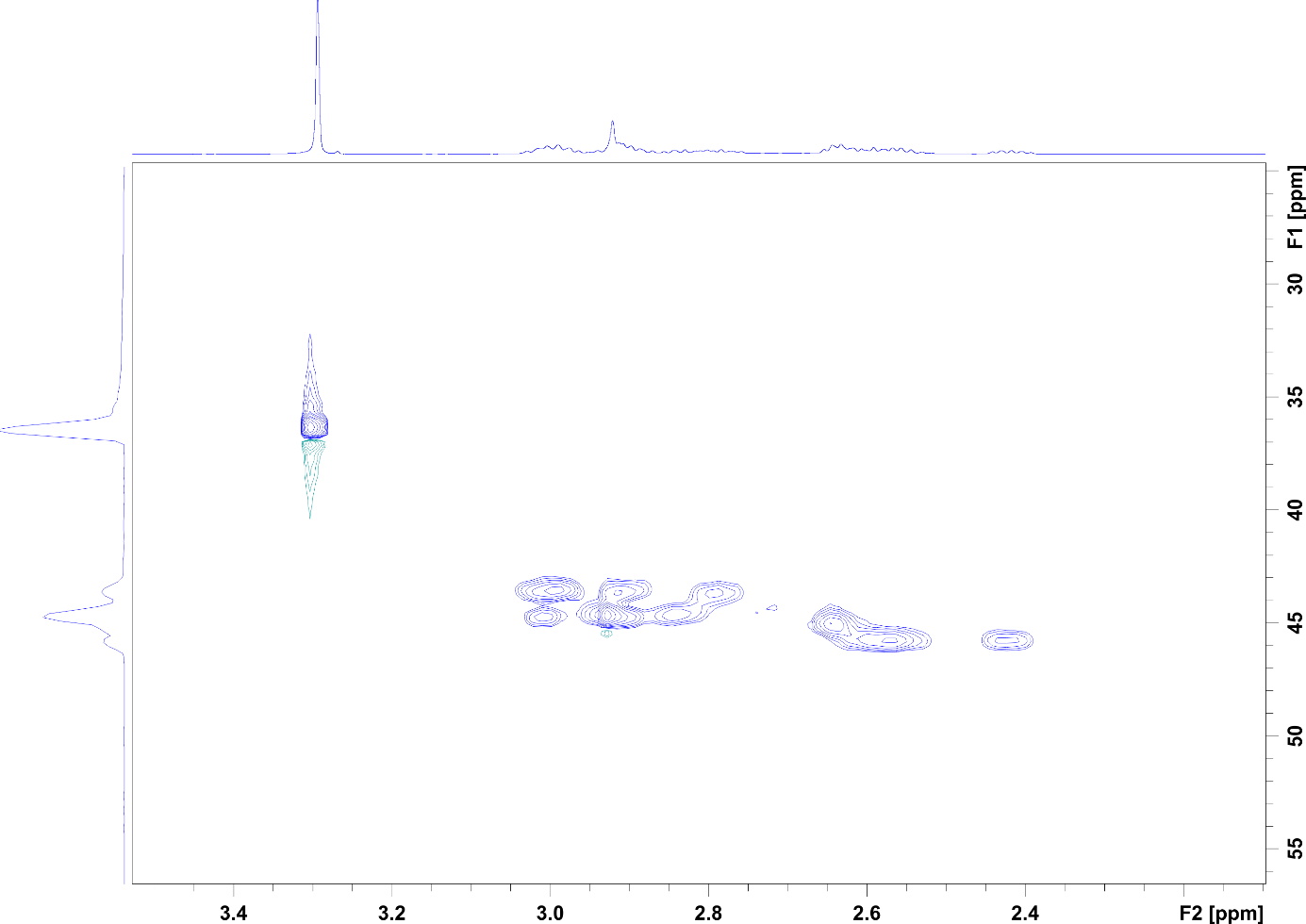
**

**Figure S27.** The main fragment of the HSQC spectrum of compound **(2)** indicated the C and H coupling only registered in D_2_O at 298K. The presence of two isomers of (**2**) was identified in the solution.


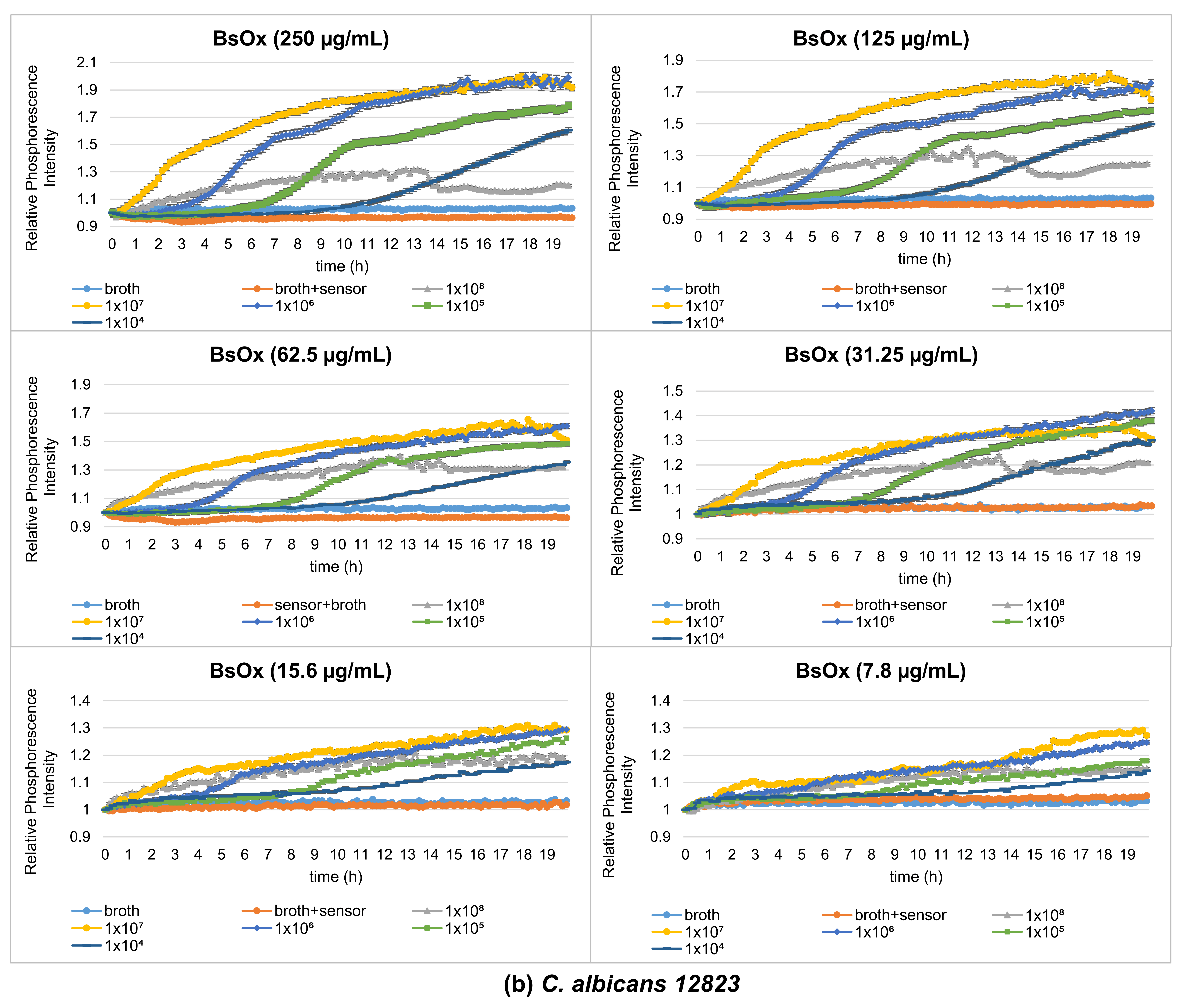

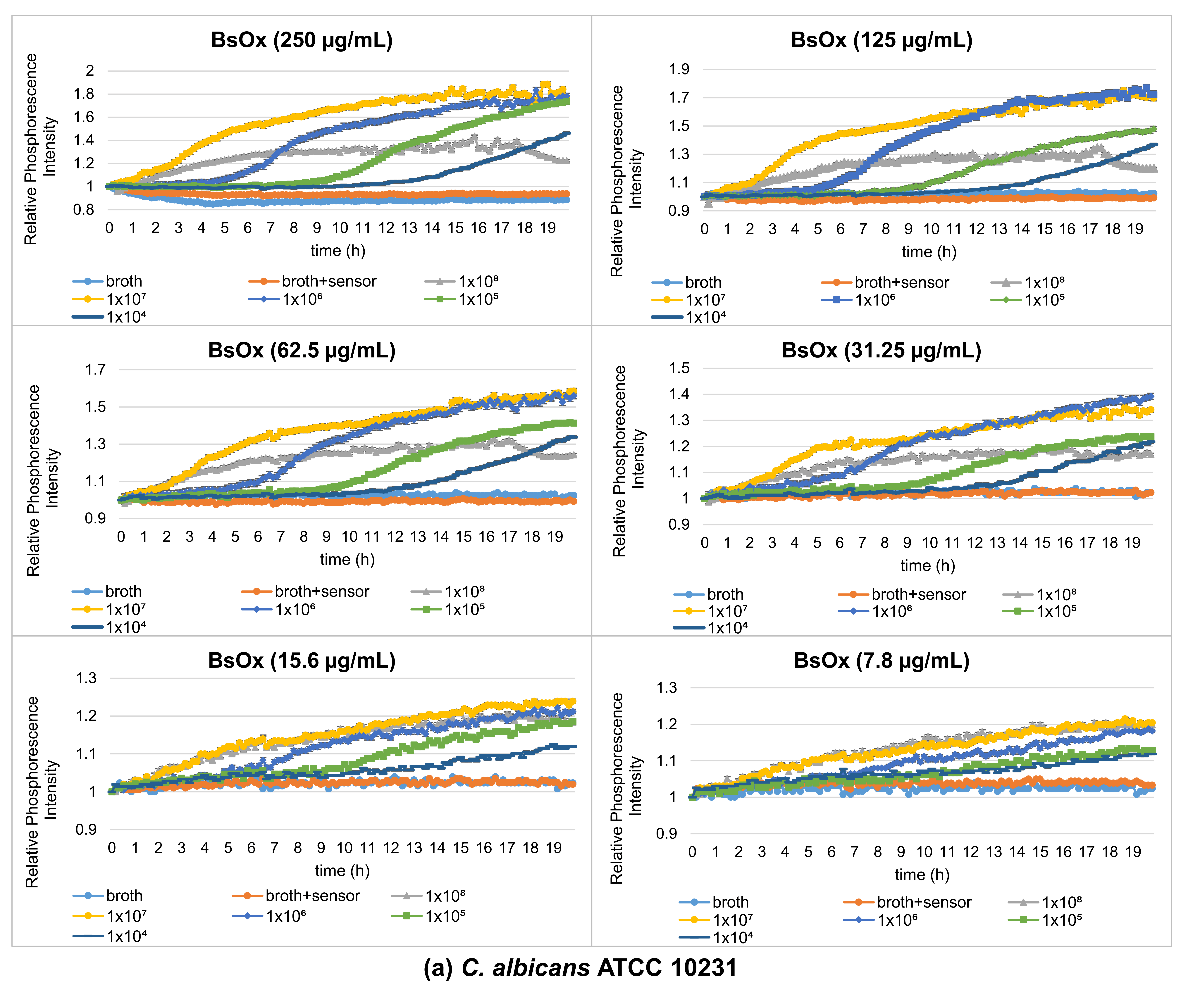
**Figure S28**. Profiles of relative phosphorescence intensities of **(a)** *C. albicans* ATCC 10231 and **(b)** *C. albicans* 12823 strains, against time for different concentrations of Box sensor (250 – 7.8 µg/mL). The optical density of the tested yeast was 10^7^ CFU/mL.


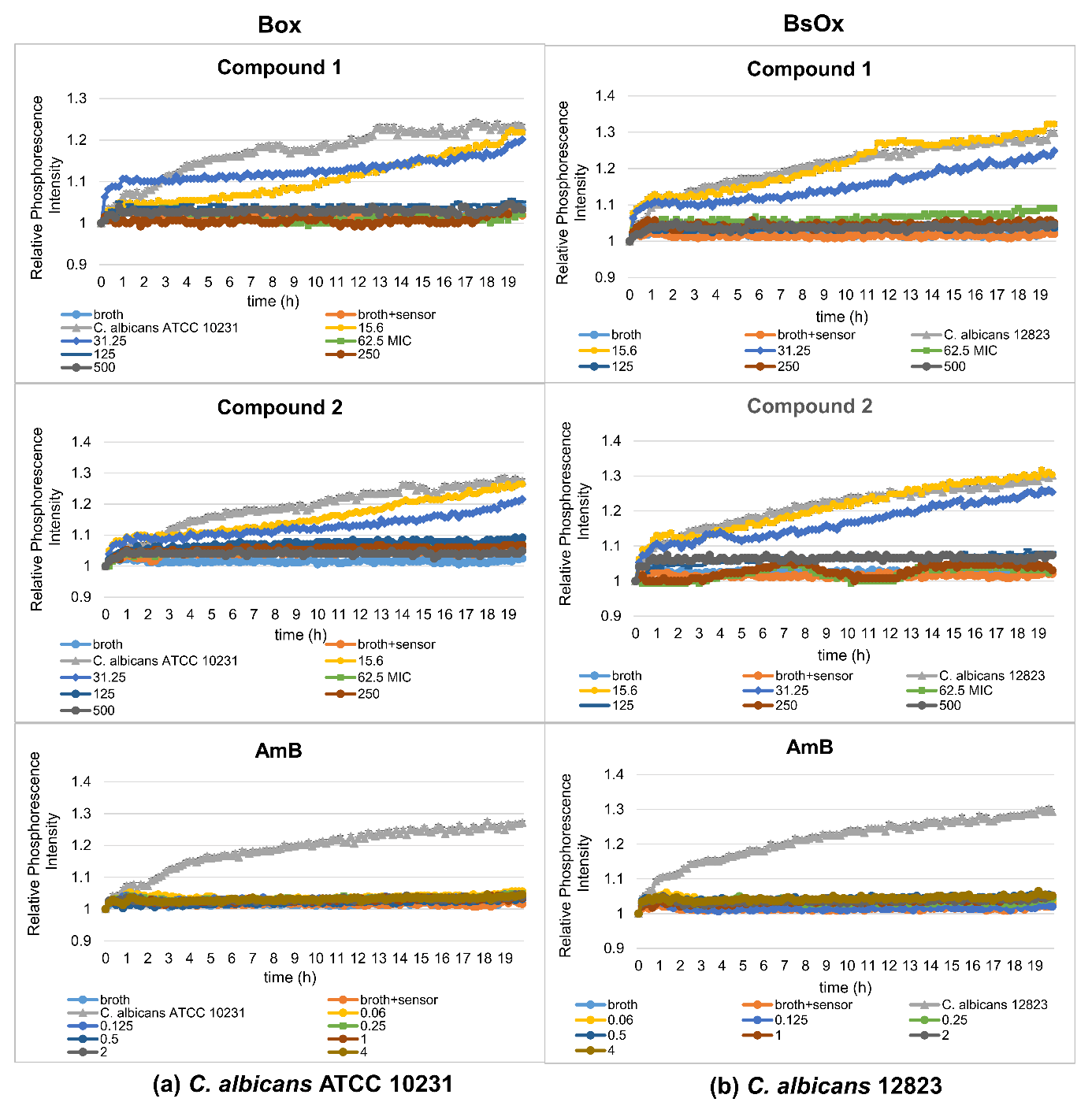


**Figure S29*.*** Profiles of relative phosphorescence intensity of **(a)** *C. albicans* ATCC 10231 and **(b)** *C. albicans* 12823 strains against different concentrations of compound (**1**), compound (**2**)**,** and AmB with Box and BsOx (31.25 µg/mL) sensors. In the legend, the sample concentration is expressed in µg/mL.


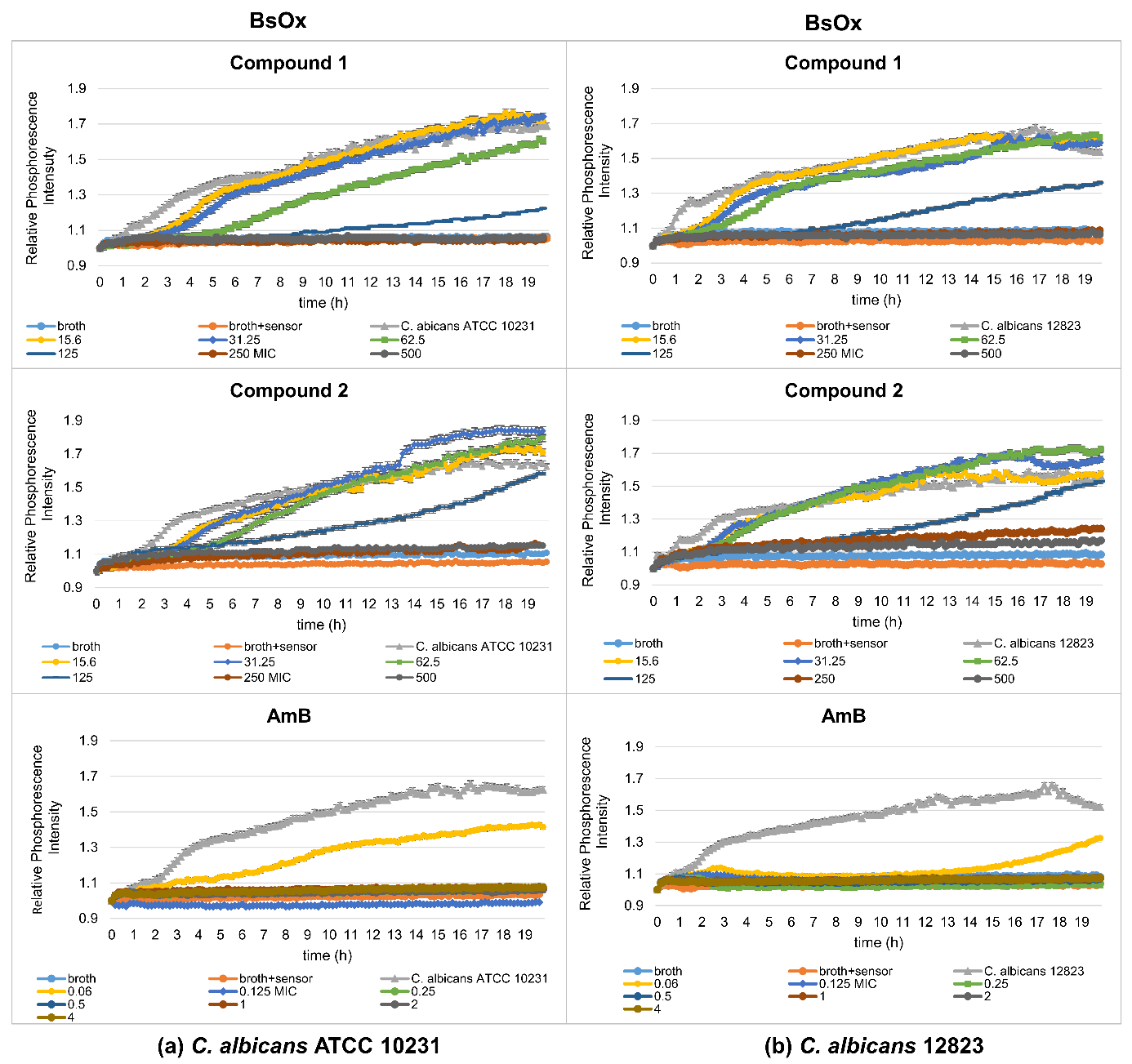


**Figure S30.** Profiles of relative phosphorescence intensities of **(a)** *C. albicans* ATCC 10231 and **(b)** *C. albicans* 12823 strains against different concentrations of compound (**1**), compound (**2**)**,** and AmB with Box and BsOx (62.5 µg/mL) sensors. In the legend, the sample concentration is expressed in µg/mL.


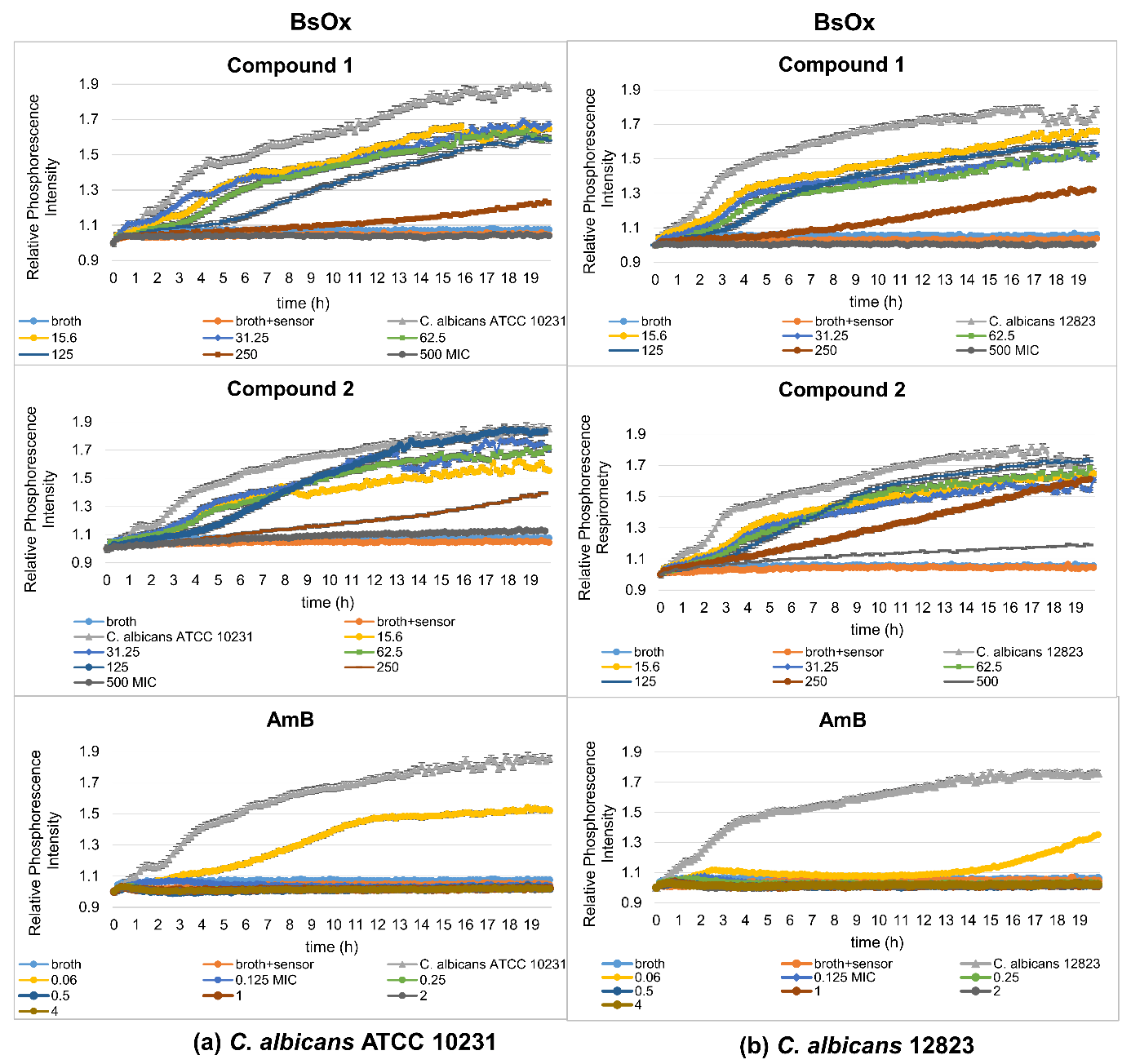


**Figure S31.** Profiles of relative phosphorescence intensities of **(a)** *C. albicans* ATCC 10231 and (b) *C. albicans* 12823 strains against the different concentrations of compound (**1**), compound (**2**)**,** and AmB with Box and BsOx (125 µg/mL) sensors. In the legend, the sample concentration is expressed in µg/mL.
