# Supplementary material for: Ru(II) Oxygen Sensors for Co(III) Complexes and Amphotericin B Antifungal Activity Detection by Phosphorescence Optical Respirometry
Source: Int J Mol Sci. 2023 May 14;24(10):8744. doi: 10.3390/ijms24108744 (PMC10218578; doi:10.3390/ijms24108744)
Supplement: Supplementary file 1 [file ijms-24-08744-s001.zip › ijms-2362536-supplementary.pdf]

Supplementary Materials

Ru(II) Oxygen Sensors for Co(III) Complexes and Amphotericin B Antifungal Activity Detection by Phosphorescence Optical Respirometry

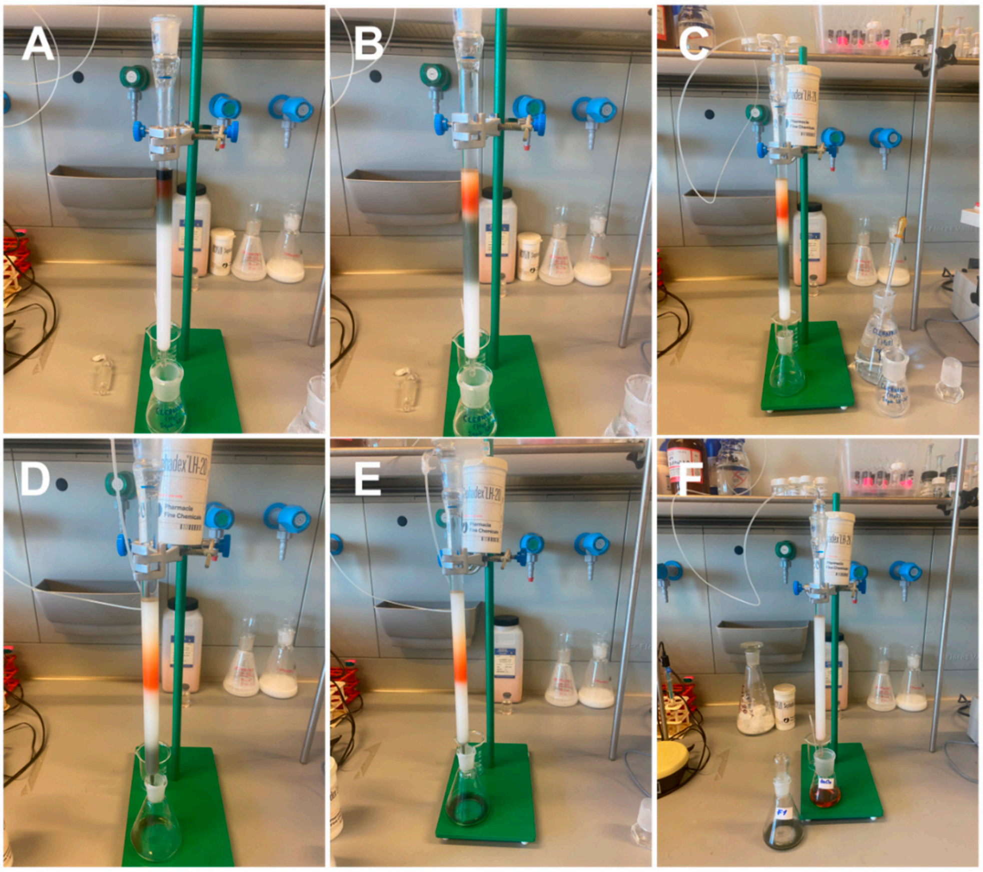

Figure S1. The $\{Ru^{II}[DPP(SO_3Na)_2]_3\}Cl_2$ coordination compound separation process on a column with Sephadex LH-20; the water was used as an eluent; the green isomer reported in the literature as well as the expected BsOx complex product - the oxygen sensor – were obtained.

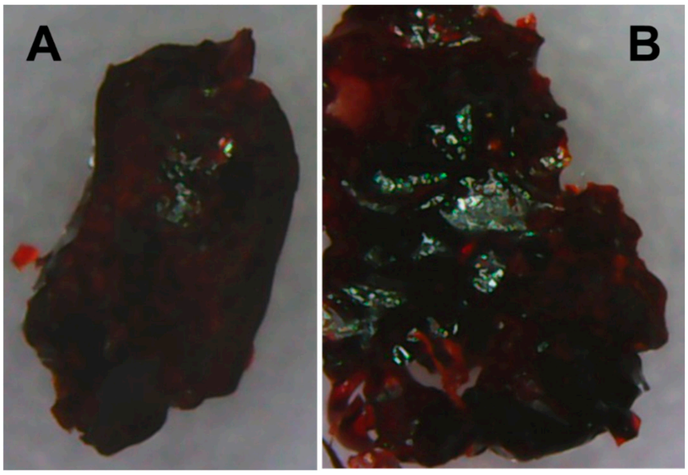

Figure S2. The microscopic pictures of the solid state of the oxygen biosensor resynthesized - {Ru[DPP(SO_3Na)$_2$]$_3$(10H_2O)}Cl_2·12H_2O: **A** and **B** present the different amorphous solid images.

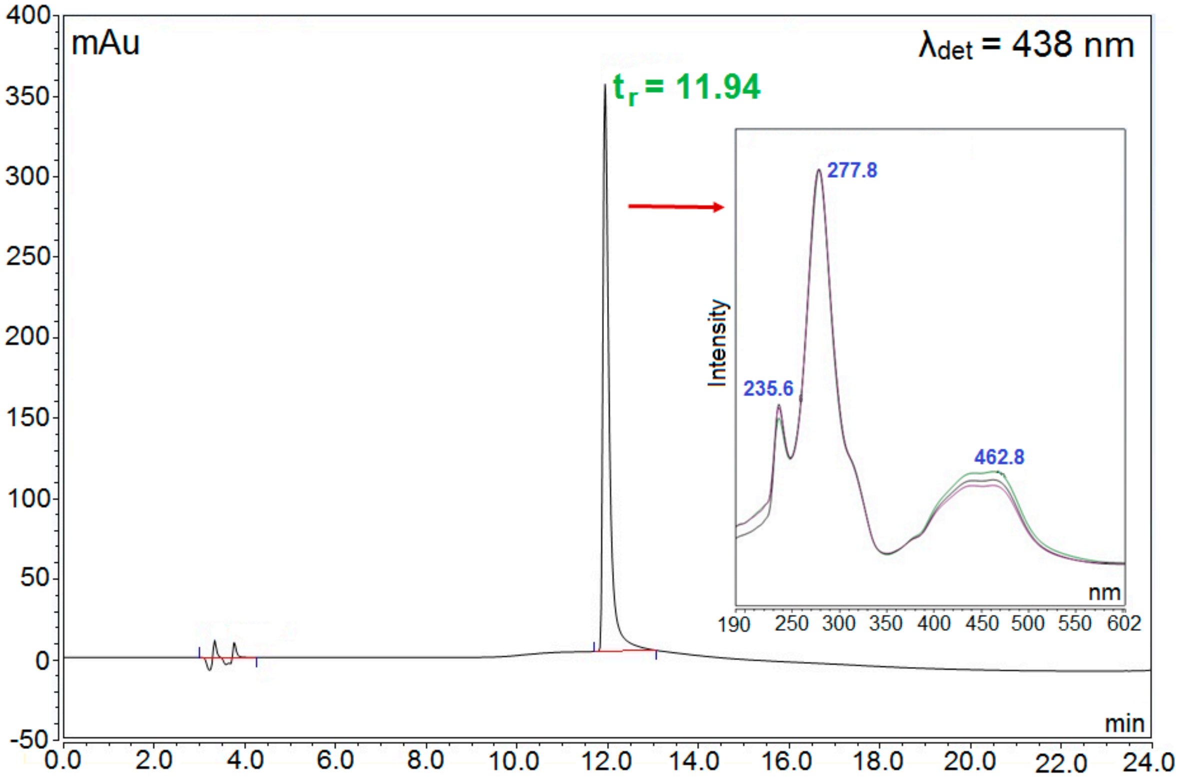

Figure S3. UHPLC chromatogram obtained (WVL-438 nm) for BsOx purity identification together with the BsOx UV-Vis spectra registered during this type of analysis.

Table S1. The data was obtained from UHPLC analyses.

Retention time [min]	Peak Area [mAu·min]	Relative Area [%]
3.30	0.1622	0.28
11.94	56.841	99.72

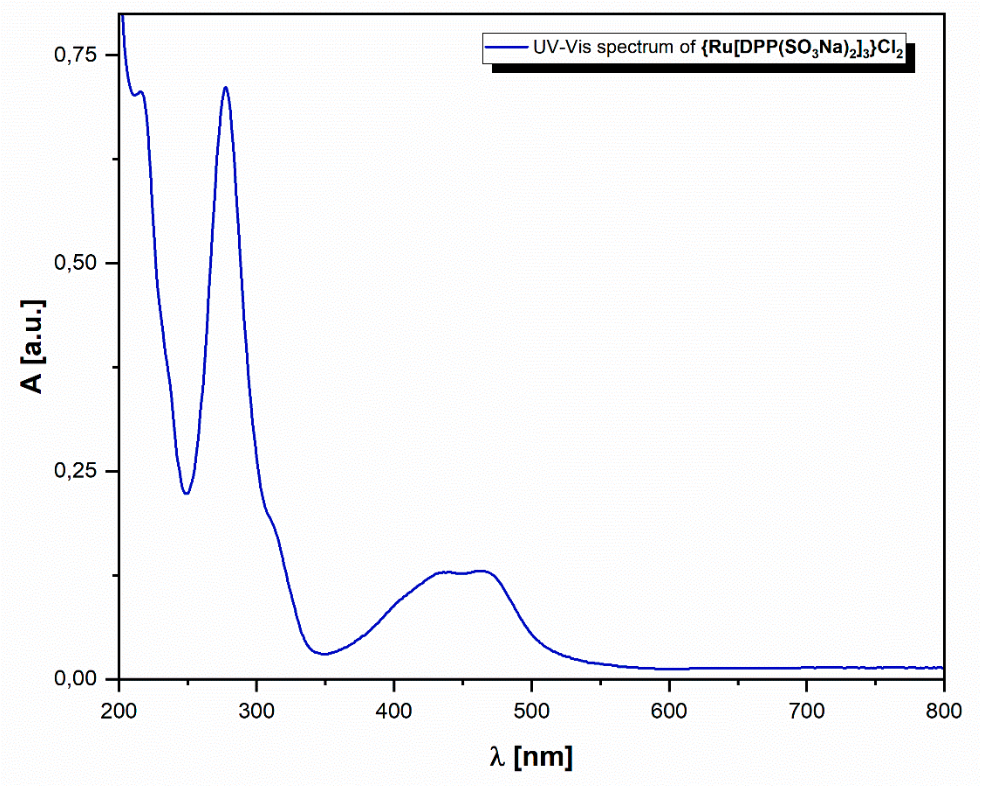

Figure S4. The absorption spectrum of an aqueous solution of $\{Ru^{II}[DPP(SO_3Na)_2]_3\}Cl_2$ studied.

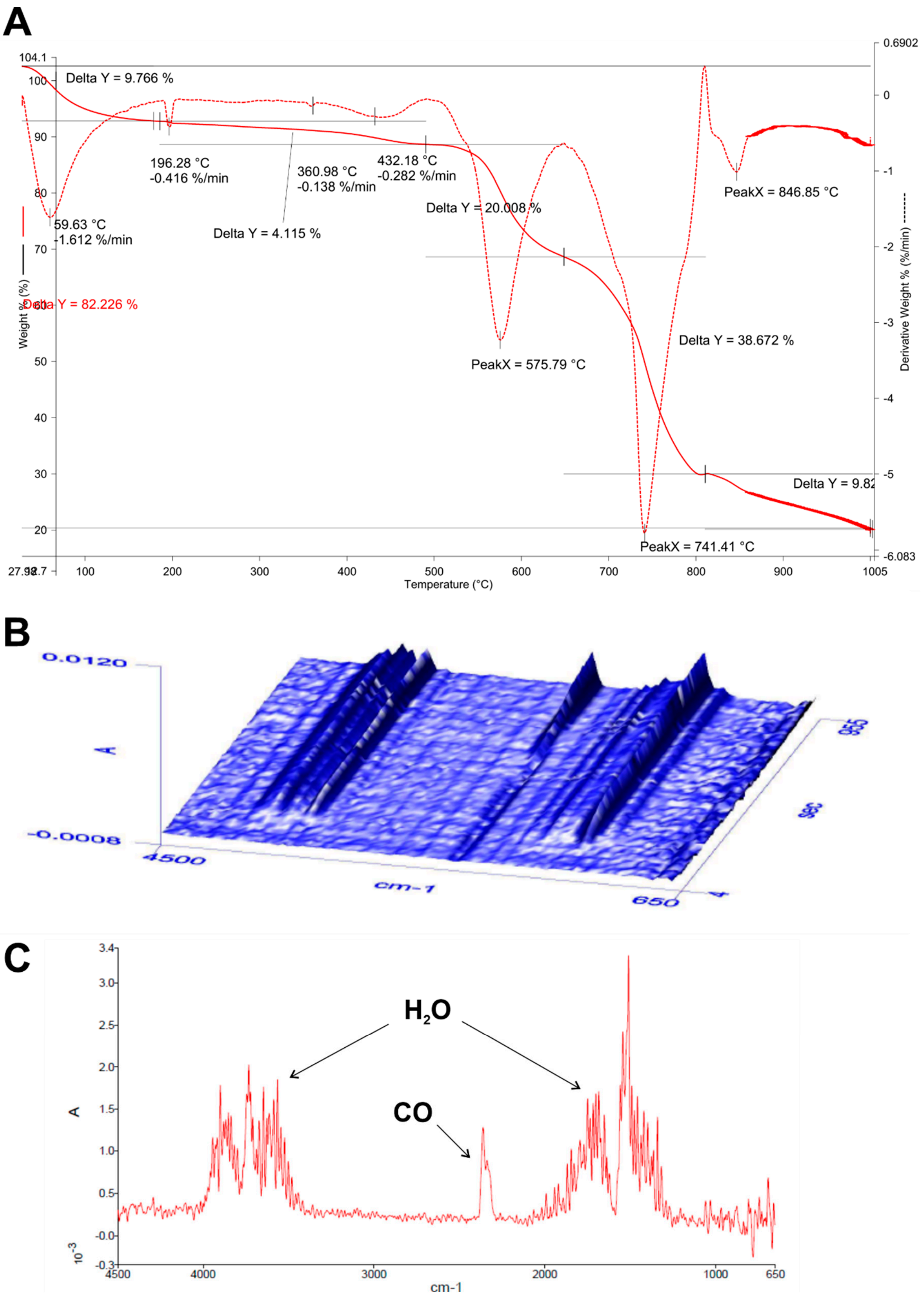

Figure S5. The hydration state establishing by using TG/IR measurements: **A.** the subsequent loss of BsOx complex sample weight registered; the first step of decomposition is the evidence of the BsOx hydrate form of solid received; **B.** the presentation of the 3D spatial IR spectra fragment of gaseous products generated by TG measurement; **C.** IR spectra of H_2O and CO emitted from BsOx complex in 704.56 s of TG/IR analysis; the results presented in **B** and **C** were obtained for 1.607 mg of BsOx sample weight.

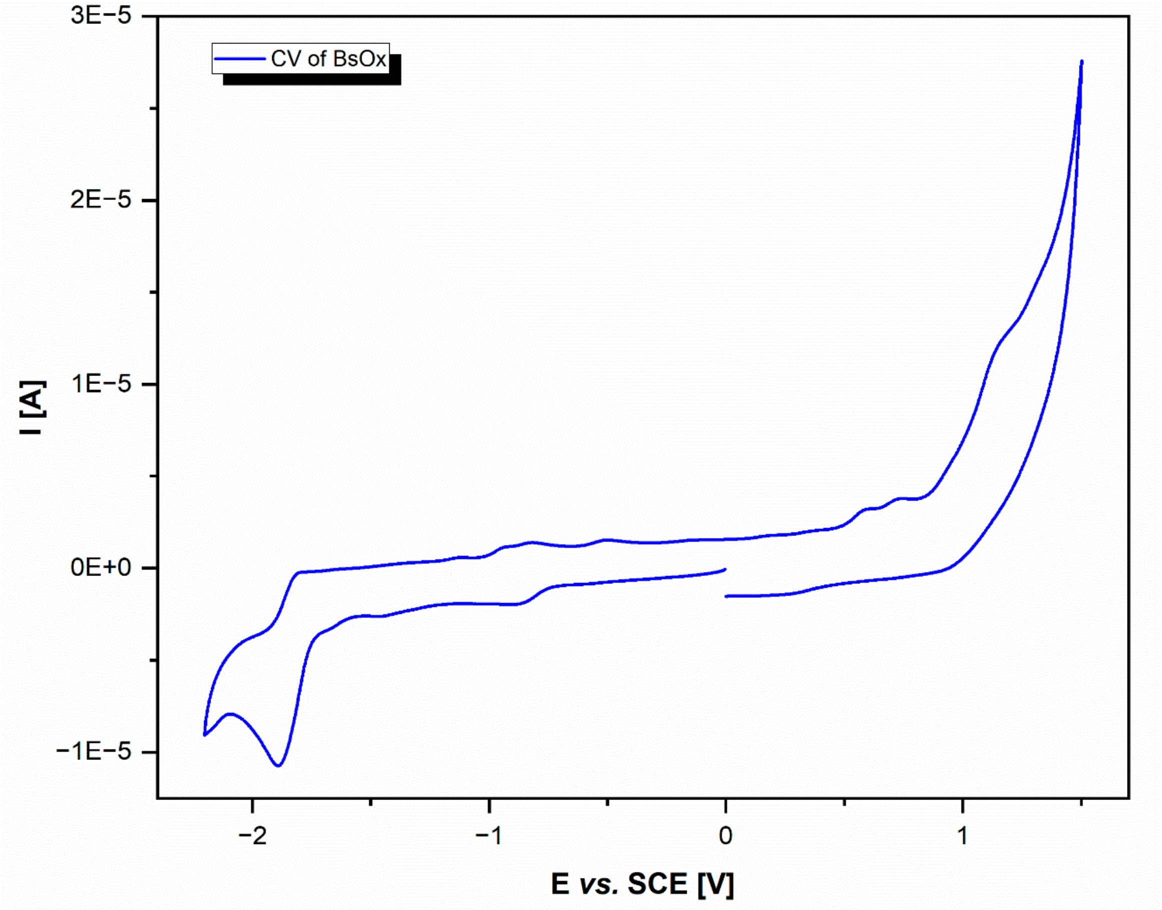

Figure S6. Voltamperometric curve of DMSO solution of the BsOx complex (10^{-3} M with addition of the standard electrolyte for the non-aqueous medium, 0.1 M TBAP) recorded by using glassy carbon working electrode (Ø = 2 mm GCE, scan rate: 100 mV/s).

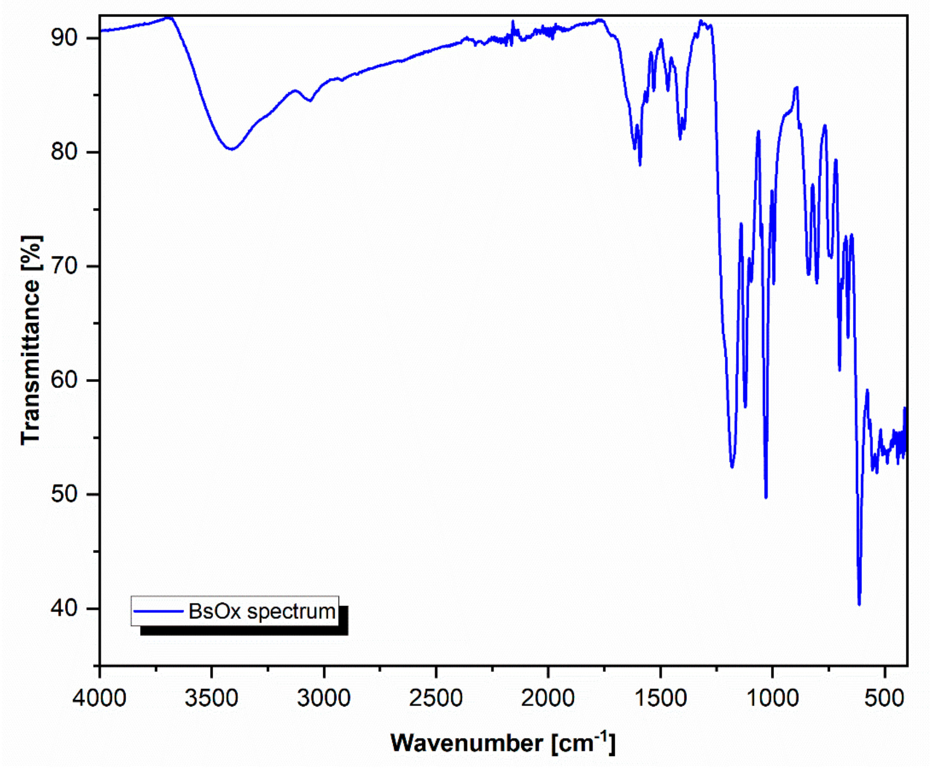

Figure S7. ATR spectrum of ruthenium(II) complex synthesized. The BsOx solid sample was used to register the oscillatory vibration bands.

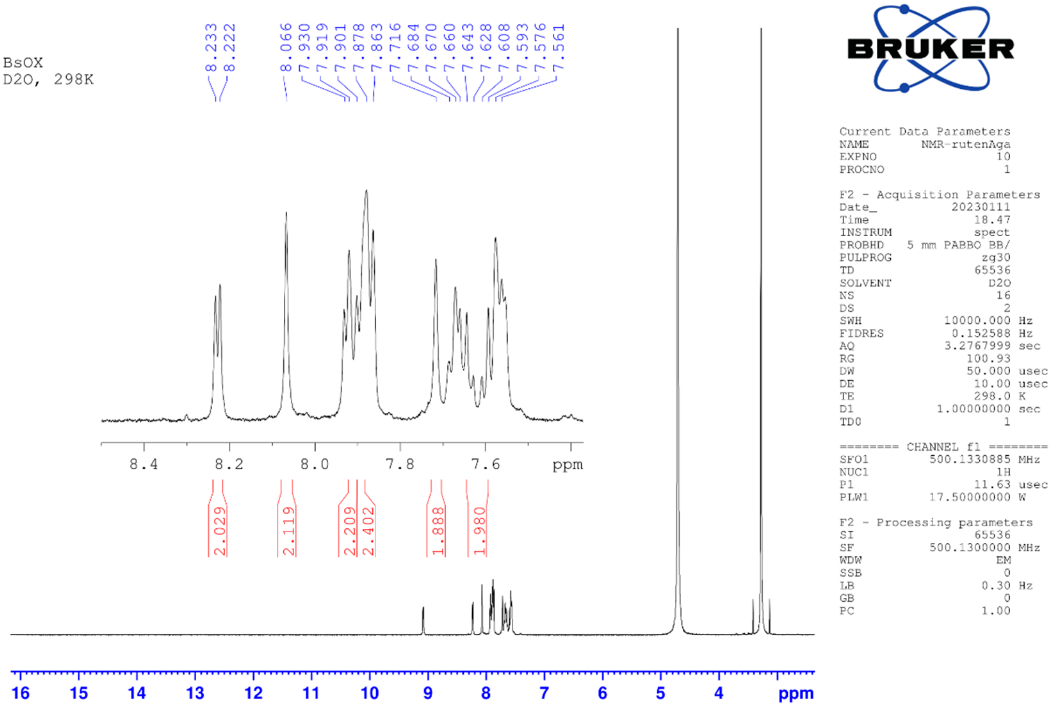

Figure S8. ^{1}H NMR spectrum of BsOx with proton integration for {Ru[L]$_{1/3}$}Cl_2 (where L: $C_{24}H_{14}N_2Na_2O_6S_2{\cdot}3H_2O$) registered in D_2O at 298K. The complete description was included in the manuscript's main text (see the 2.2. section);

the signals at 4.69 ppm for D_2O as a solvent; 3.27 ppm for H_2O hydrate as well as the typical H_2O signal positions associated with D_2O.

A

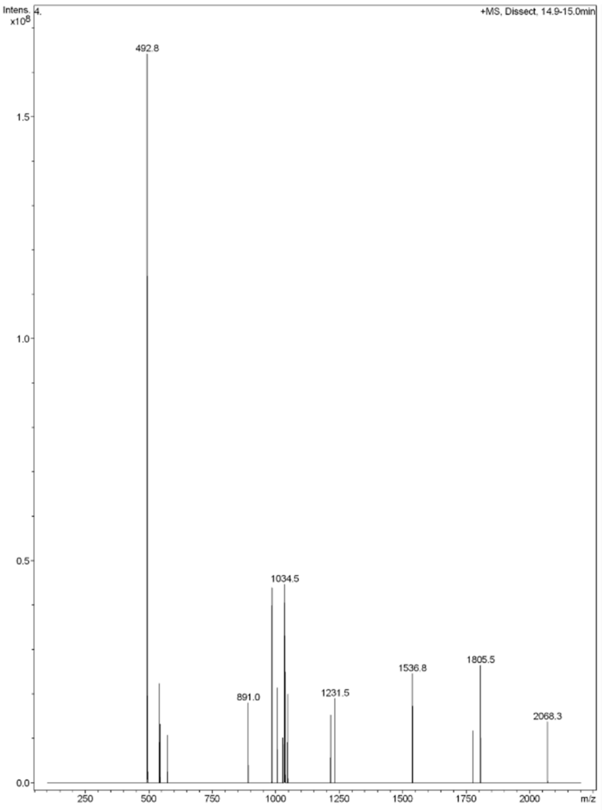

B

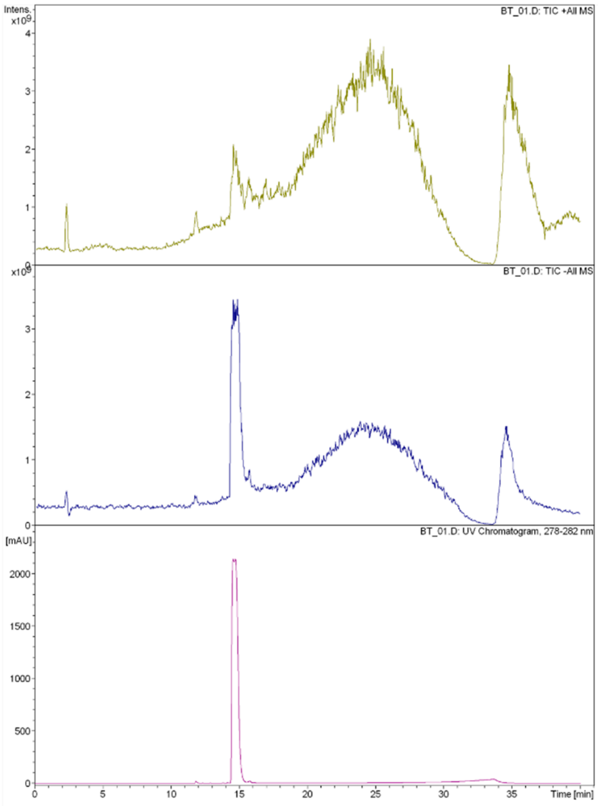

C

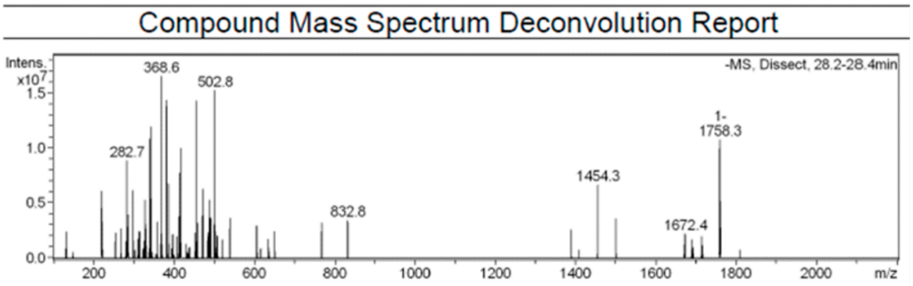

Component	Molecular Mass	Molecule		Absolute Abundance	Relative Abundance
A	1757.5	1758.5	[M - H]-	10713268	100.00

Component A Detail

Actual Peak	Charge	Isotopic Mass([M - H]-)	Predicted Peak
1757.7	1-	1757.7	1757.5
1758.3	1-	1758.3	1758.5
1759.2	1-	1759.2	1759.6
1760.1	1-	1760.1	1760.6

Molecular Mass ([M - H]-): 1757.5 Std. Deviation: 0.318477
Average Mass ([M - H]-): 1758.5

Figure S9. The LCMS results for BsOx registered after 15 minutes **A.** The m/z peaks were observed for multiple ionization of the compound. **B.** chromatograms: top – the water background; middle - compound together with the background; bottom – the aqueous solution signal for pure BsOx complex; the background is excluded (15 minutes); **C.** the selected MS negative mode mass spectrum of BsOx included in the deconvolution report for BsOx complex: *found:* 1758.5 [M-Na$^+$]; *calc.* 1781.5 [M].

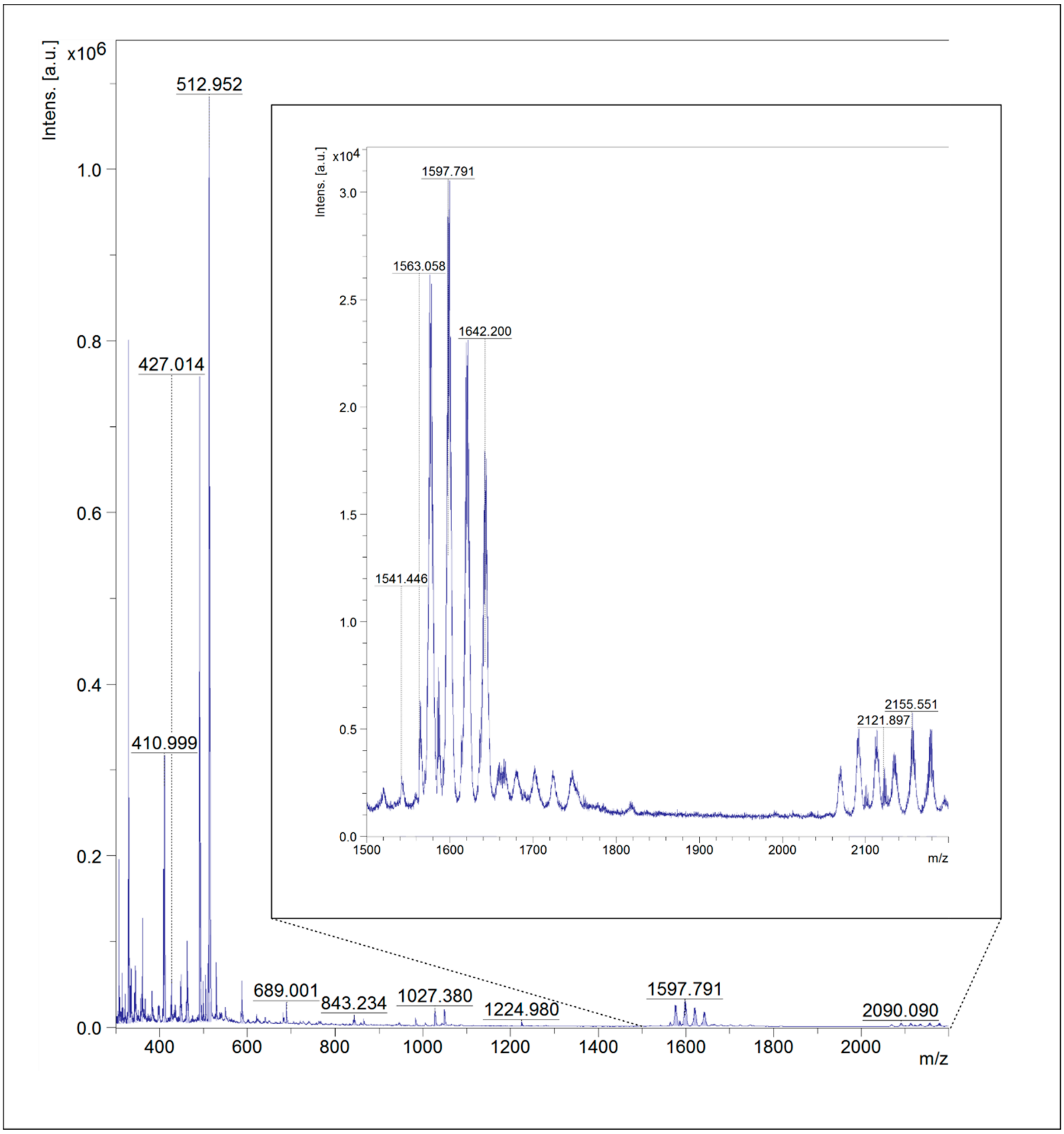

Figure S10. MALDI-TOF mass spectrum (DHB matrix) obtained for BsOx probe (multistep ionization process was confirmed by both results of analyses MALDI and QTOF, respectively).

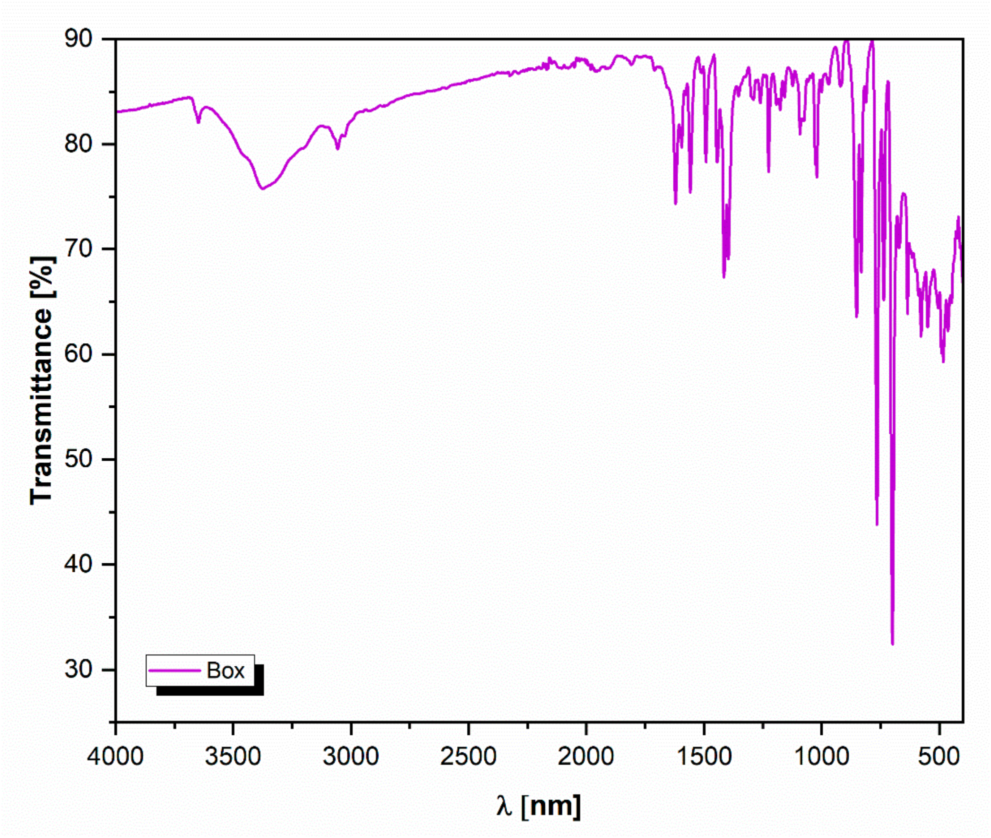

Figure S11. ATR spectrum of ruthenium(II) and DPP complex synthesized. The Box solid sample was used to register the oscillatory vibration bands.

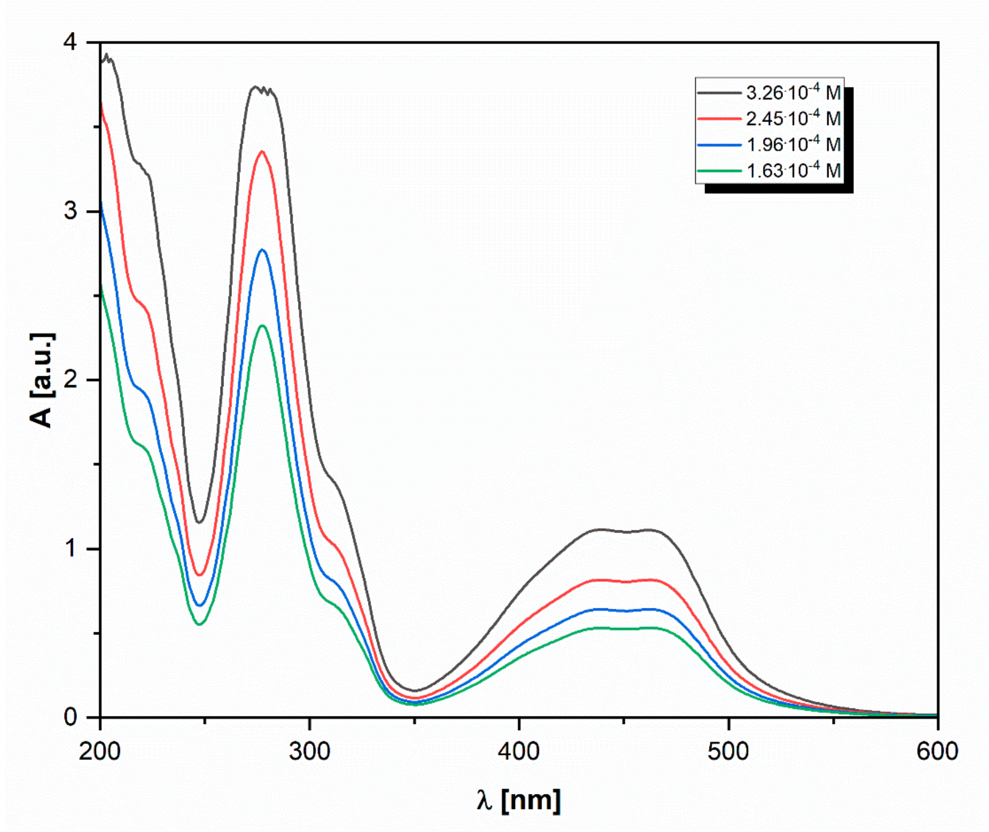

Figure S12. The absorption spectra of $[Ru^{II}(DPP)_3]Cl_2$ studied (Box) aqueous solutions with different concentrations.

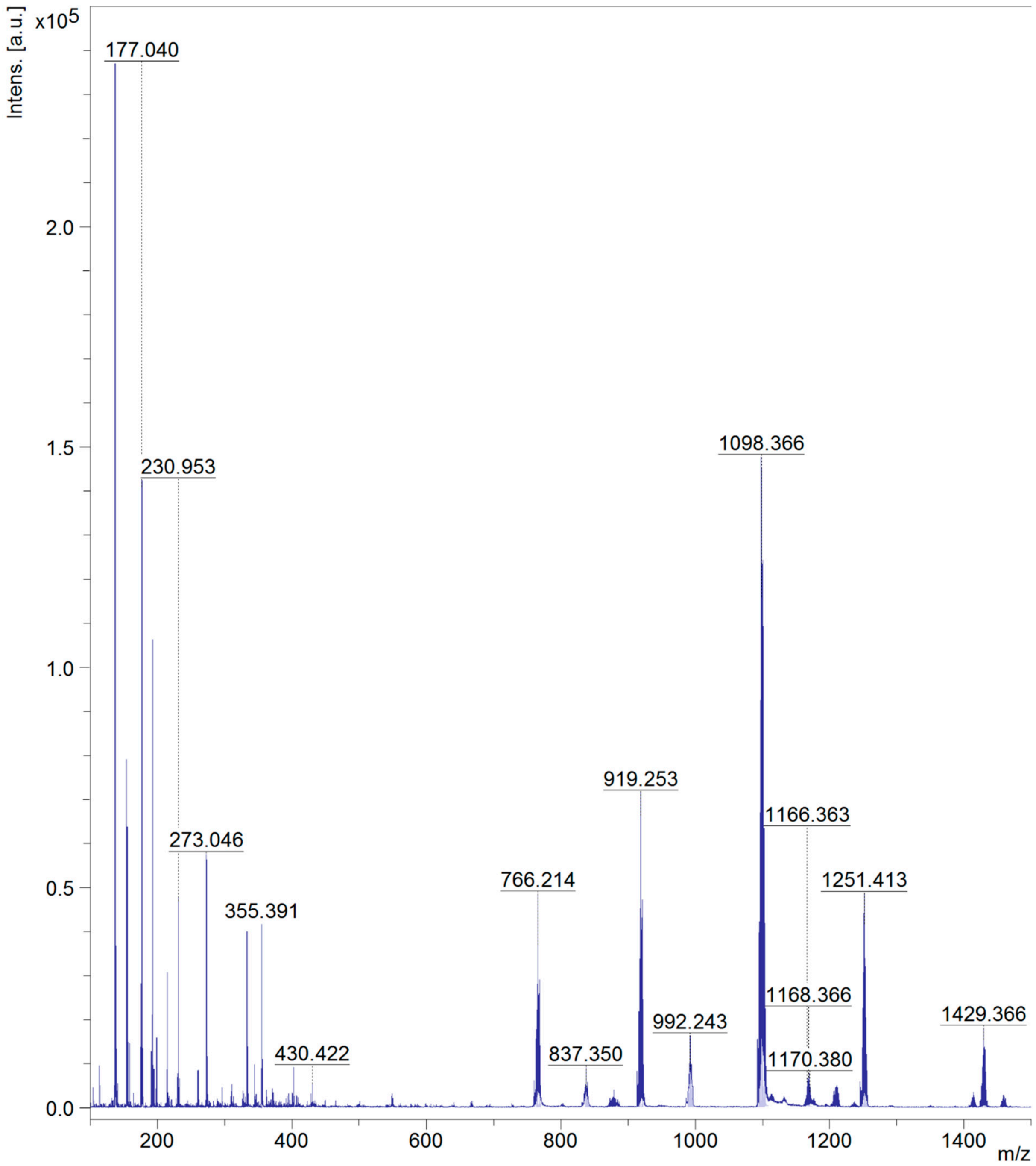

Figure S13. MALDI-TOF mass spectrum (DHB matrix) obtained for Box probe; m/z signals found (calc.): [M+H] 1170.38 (1170.24); [M-2Cl] 1098.37 (1098.24).

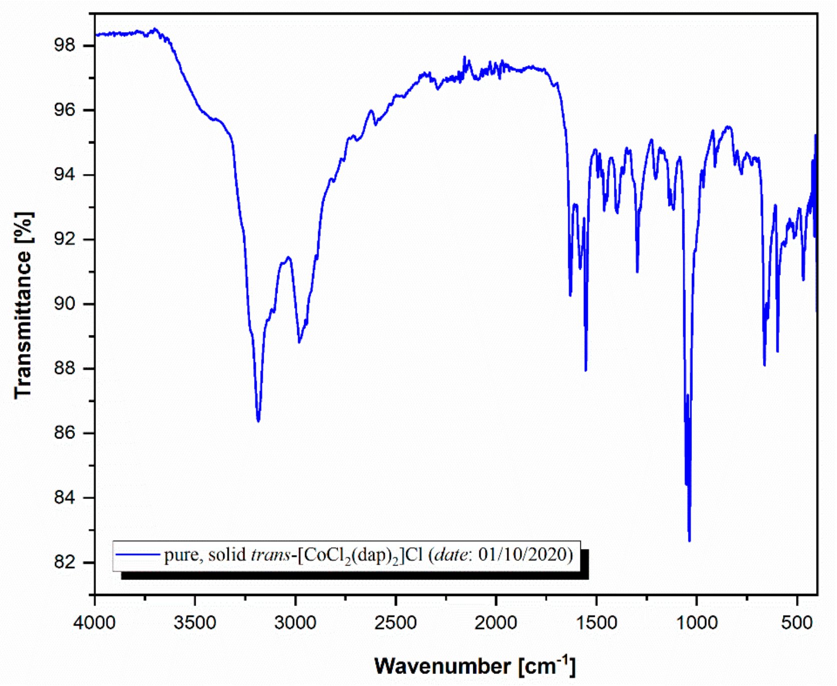

Figure S14. ATR spectrum of cobalt(III) complex synthesized with 1,3-diamine propane. The compound (**1**) solid sample was used to register the oscillatory vibration bands.

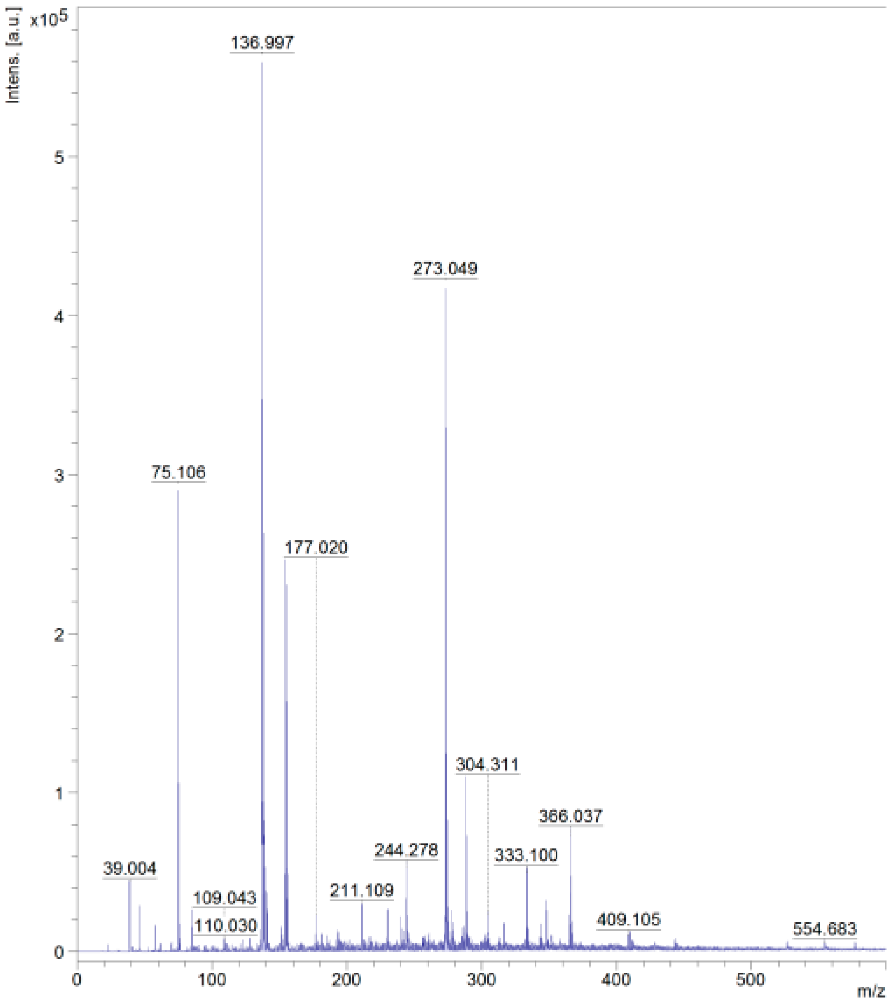

Figure S15. MALDI-TOF mass spectrum (DHB matrix) obtained for (**1**) Co(III) complex.

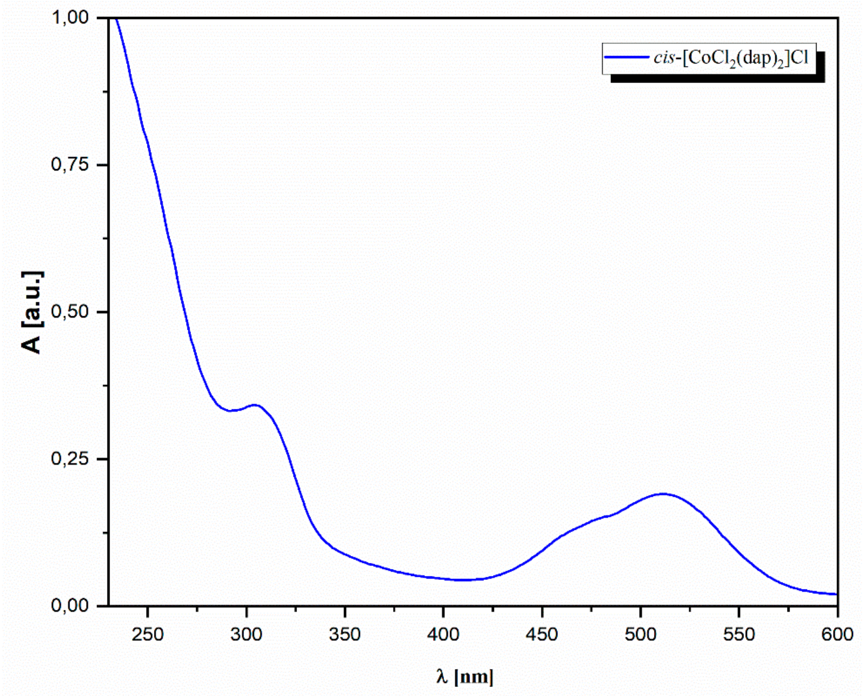

Figure S16. The absorption spectrum of an aqueous solution of coordination compound (**1**).

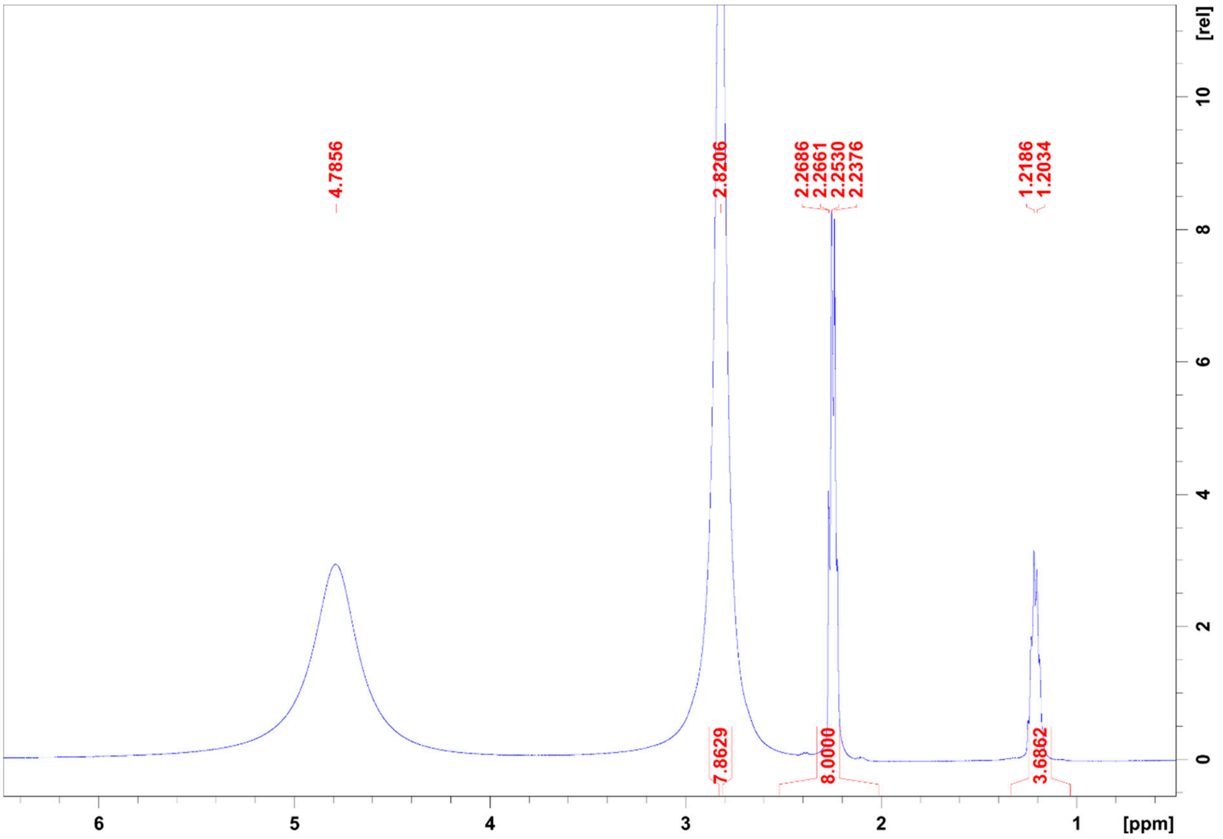

Figure S17. ^{1}H NMR spectrum of compound (**1**) with proton integration registered in D_2O at 298K.

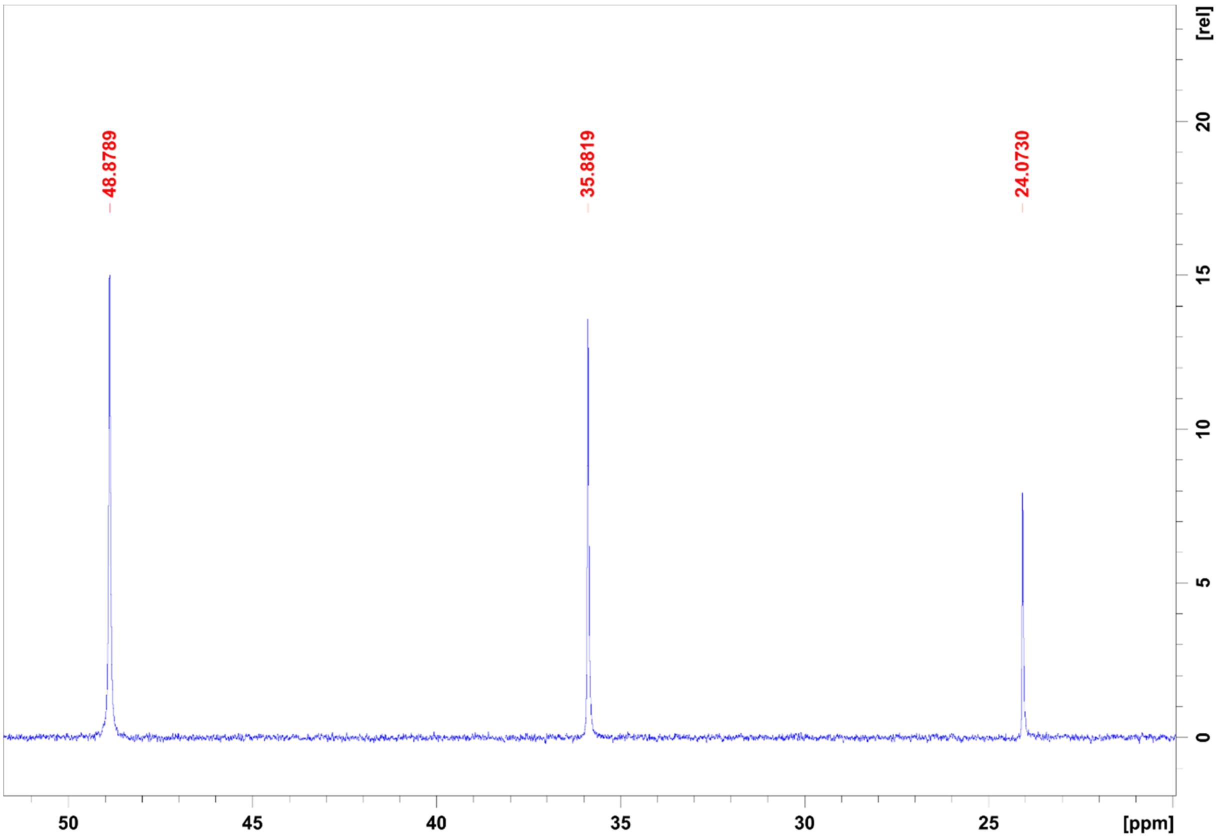

Figure S18. ^{13}C NMR spectrum of compound (**1**) together with C-signals registered in D_2O at 298K.

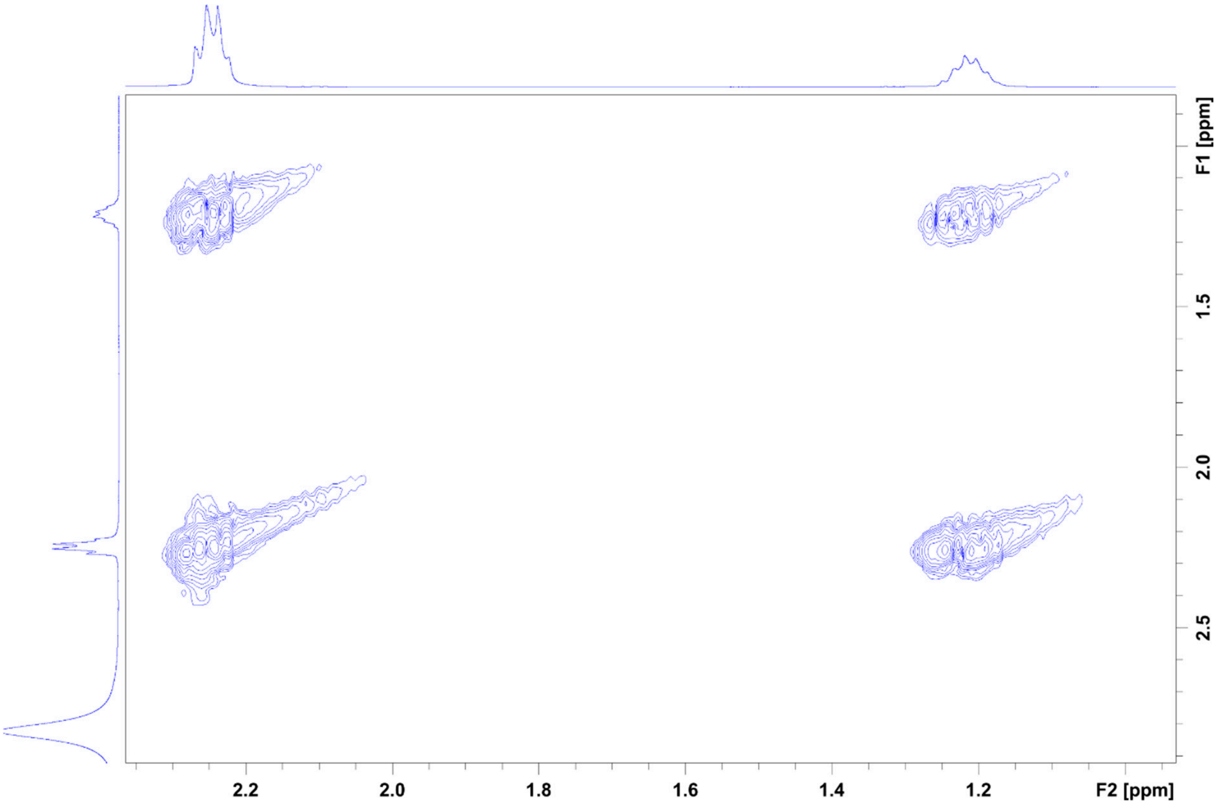

Figure S19. The 2D COSY spectrum of compound (**1**) registered in D_2O at 298K.

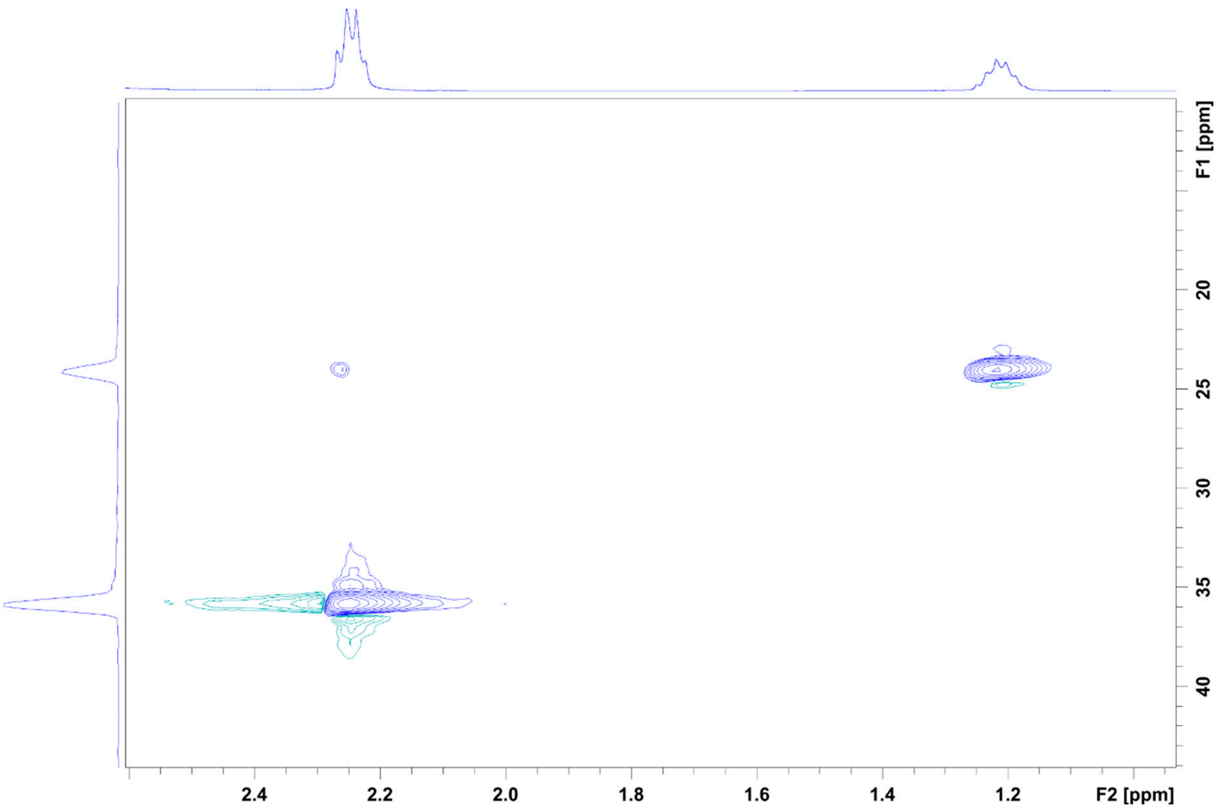

Figure S20. The main fragment of the HSQC spectrum of compound **(1)** indicated the C and H coupling only registered in D2O at 298K.

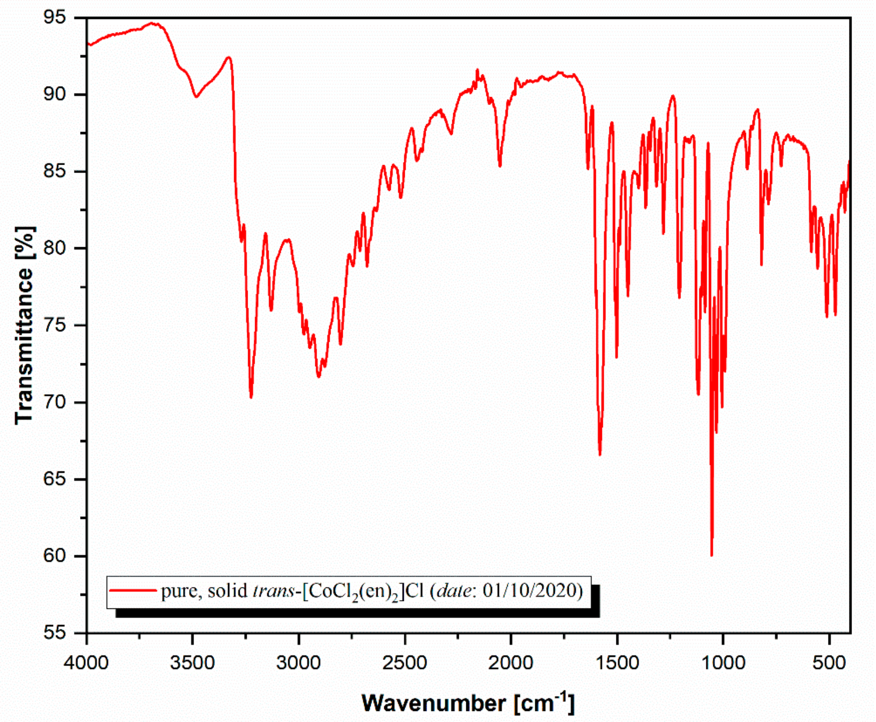

Figure S21. ATR spectrum of cobalt(III) complex synthesized with ethylenediamine. The compound (**2**) solid sample was used to register the oscillatory vibration bands.

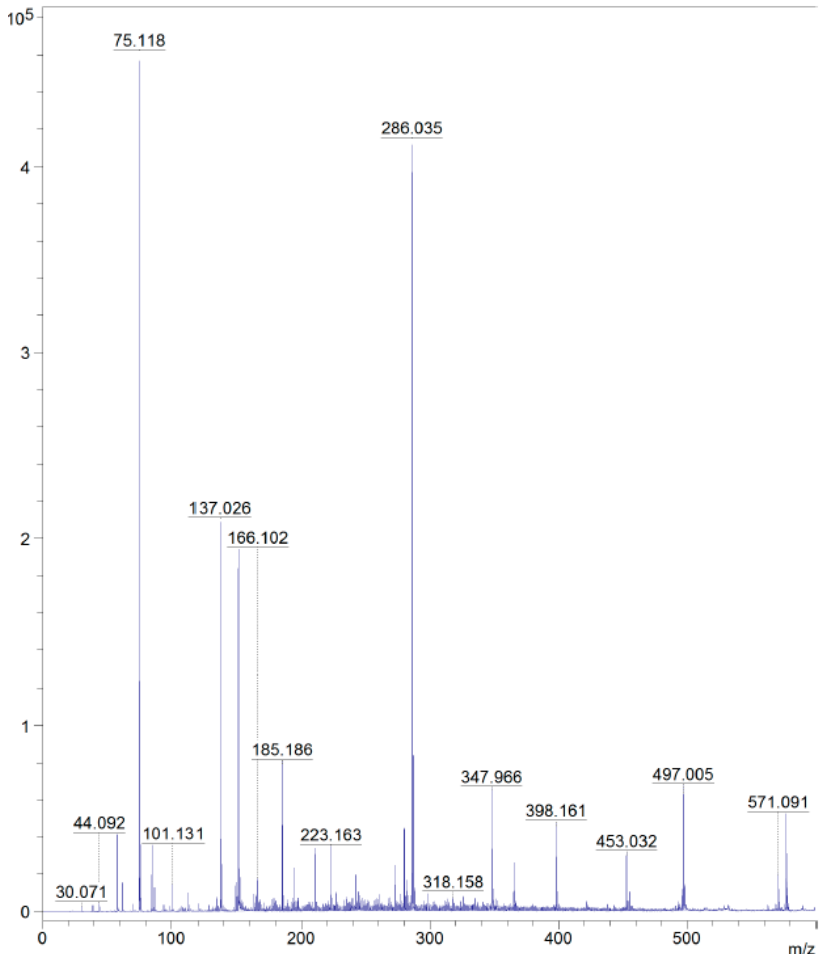

Figure S22. MALDI-TOF mass spectrum (DHB matrix) obtained for Co(III) complex (**2**).

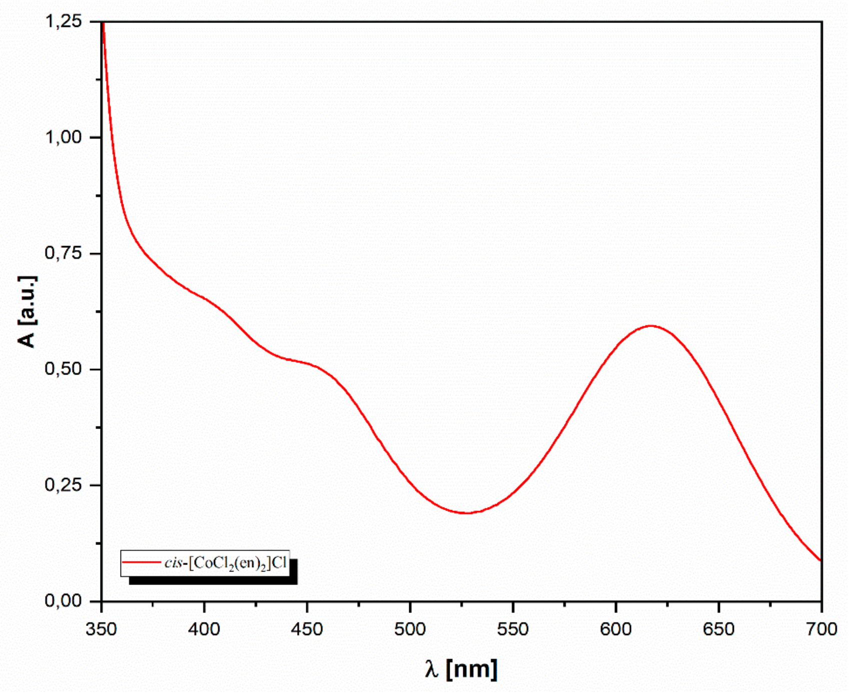

Figure S23. The absorption spectrum of an aqueous solution of coordination compound (**2**).

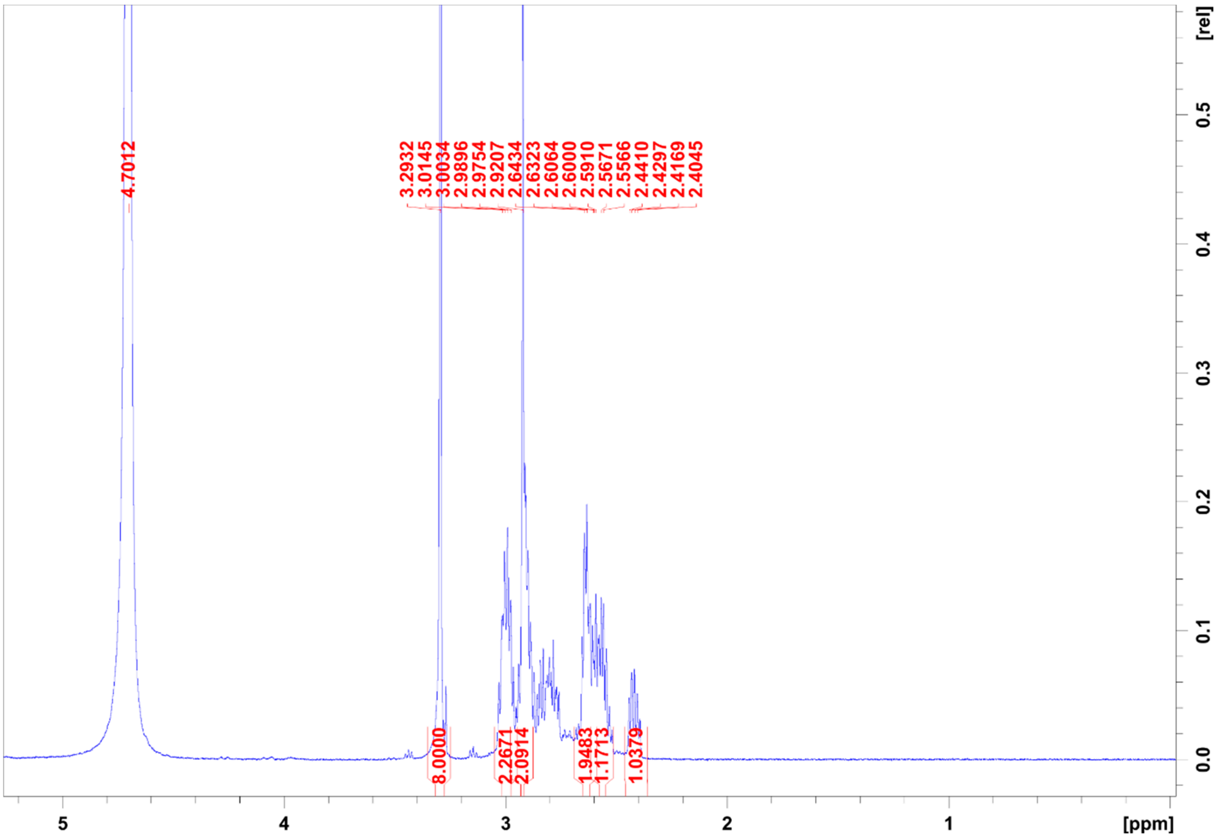

Figure S24. ^{1}H NMR spectrum of compound (**2**) with proton integration registered in D_2O at 298K.

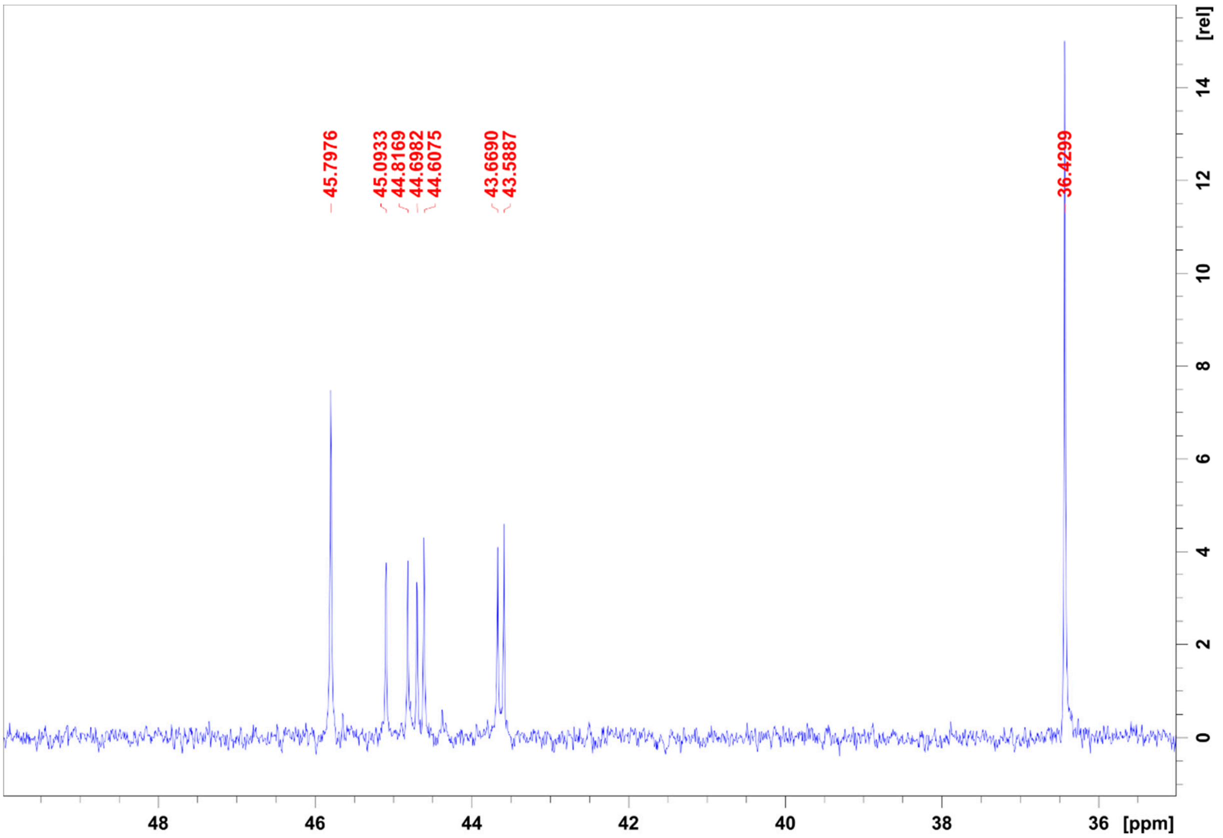

Figure S25. ^{13}C NMR spectrum of compound (**2**) together with C-signals registered in D_2O at 298K. The presence of two geometric isomers of (**2**) was identified in the solution (*cis* and *trans* form).

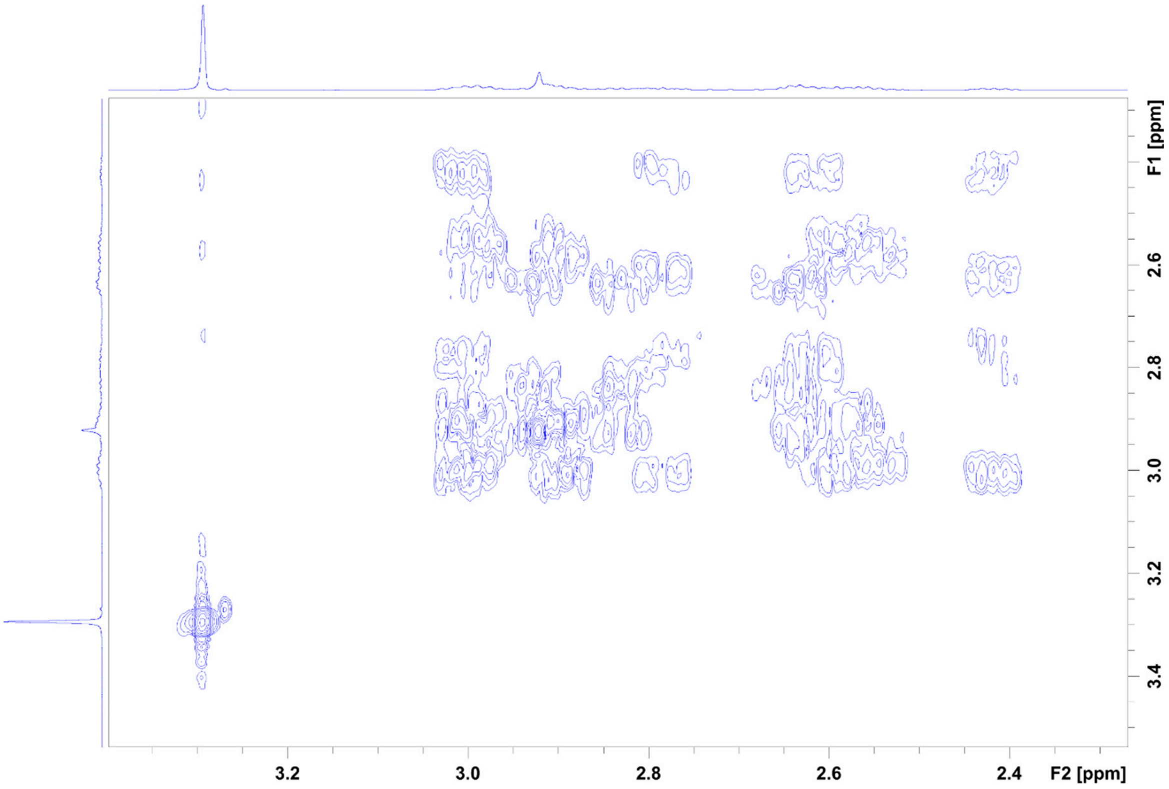

Figure S26. The 2D COSY spectrum of (**2**) is registered in D_2O at 298K. Note that the NMR identified the *cis* and *trans* forms of compound (**2**) in the D_2O.

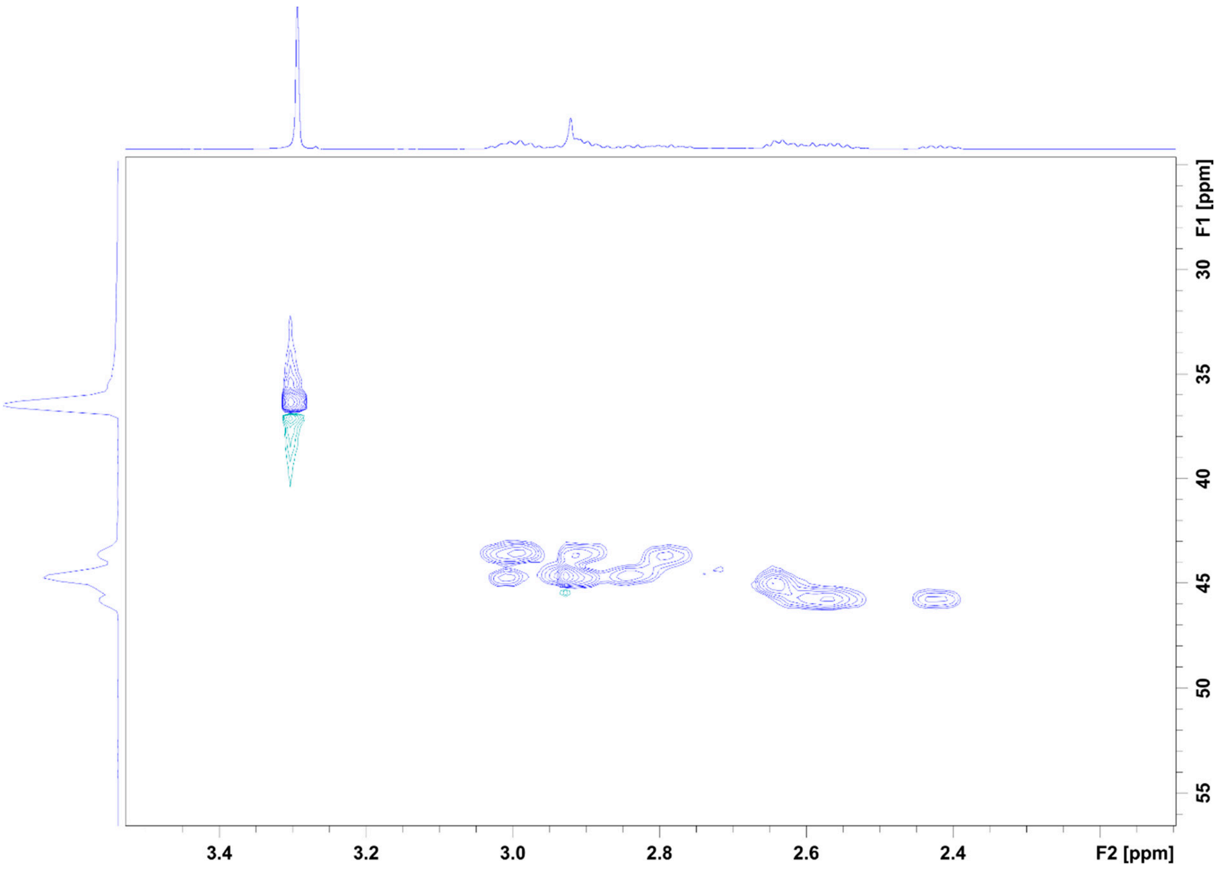

Figure S27. The main fragment of the HSQC spectrum of compound **(2)** indicated the C and H coupling only registered in D_2O at 298K. The presence of two isomers of (**2**) was identified in the solution.

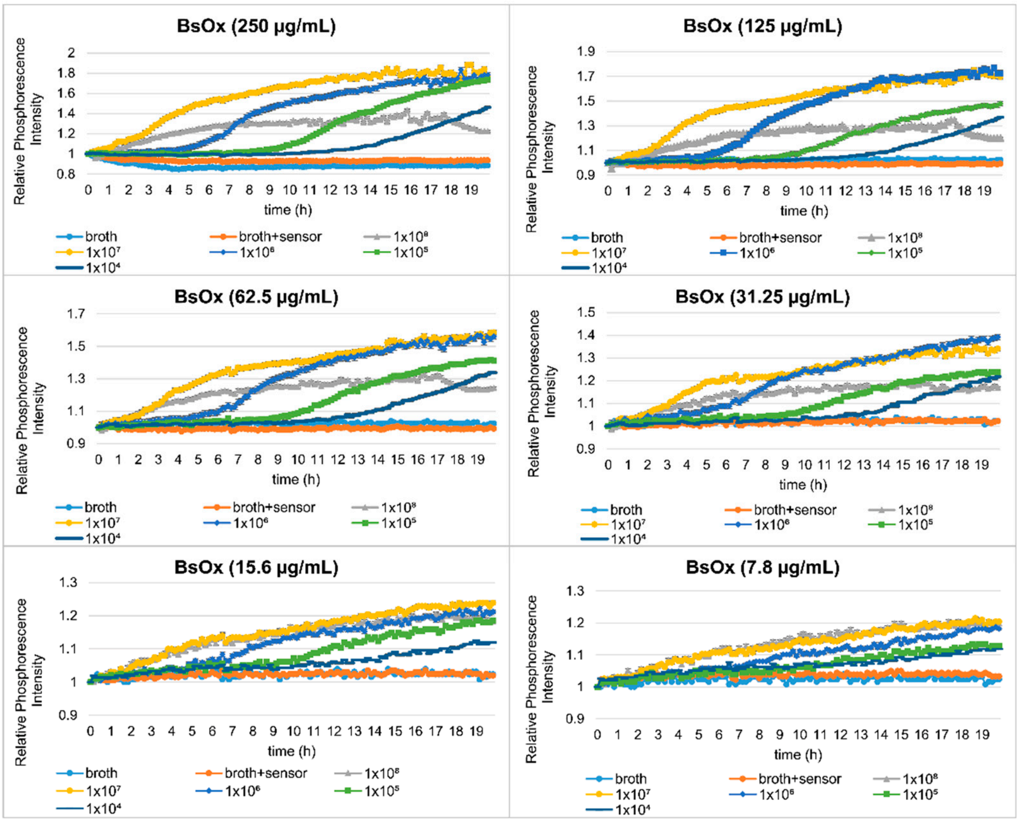

(a) *C. albicans* ATCC 10231

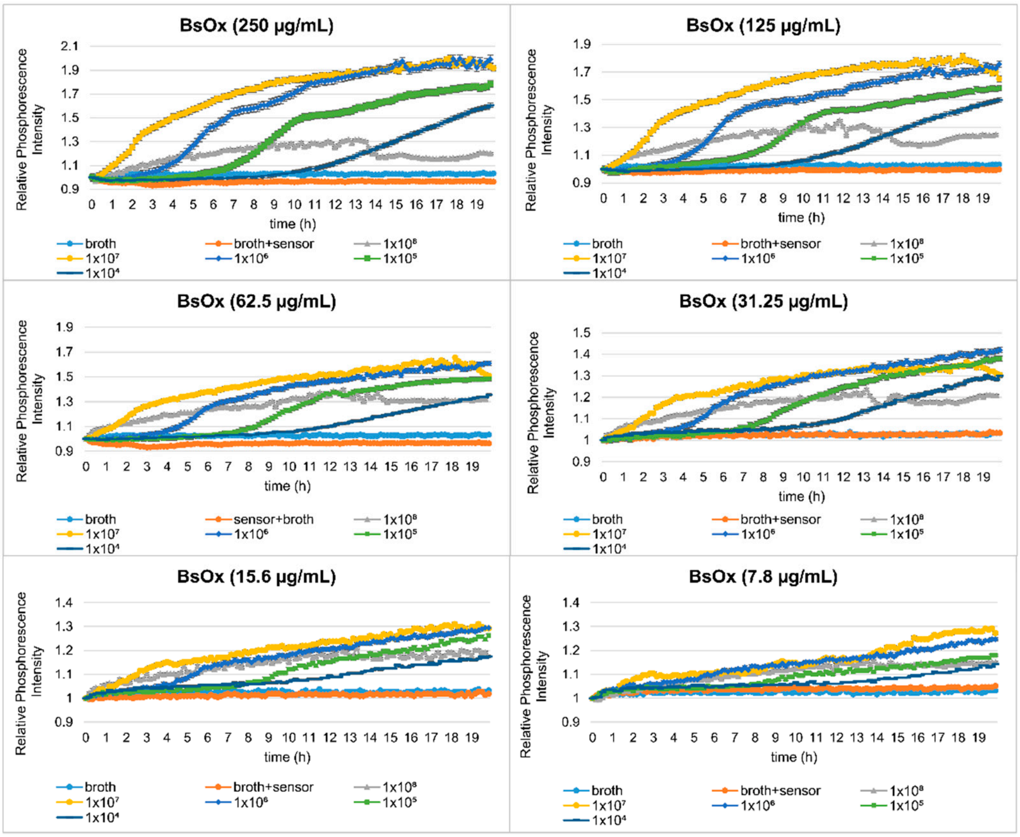

(b) *C. albicans* 12823

Figure S28. Profiles of relative phosphorescence intensities of **(a)** *C. albicans* ATCC 10231 and **(b)** *C. albicans* 12823 strains, against time for different concentrations of Box sensor (250 – 7.8 μg/mL). The optical density of the tested yeast was 10^7 CFU/mL.

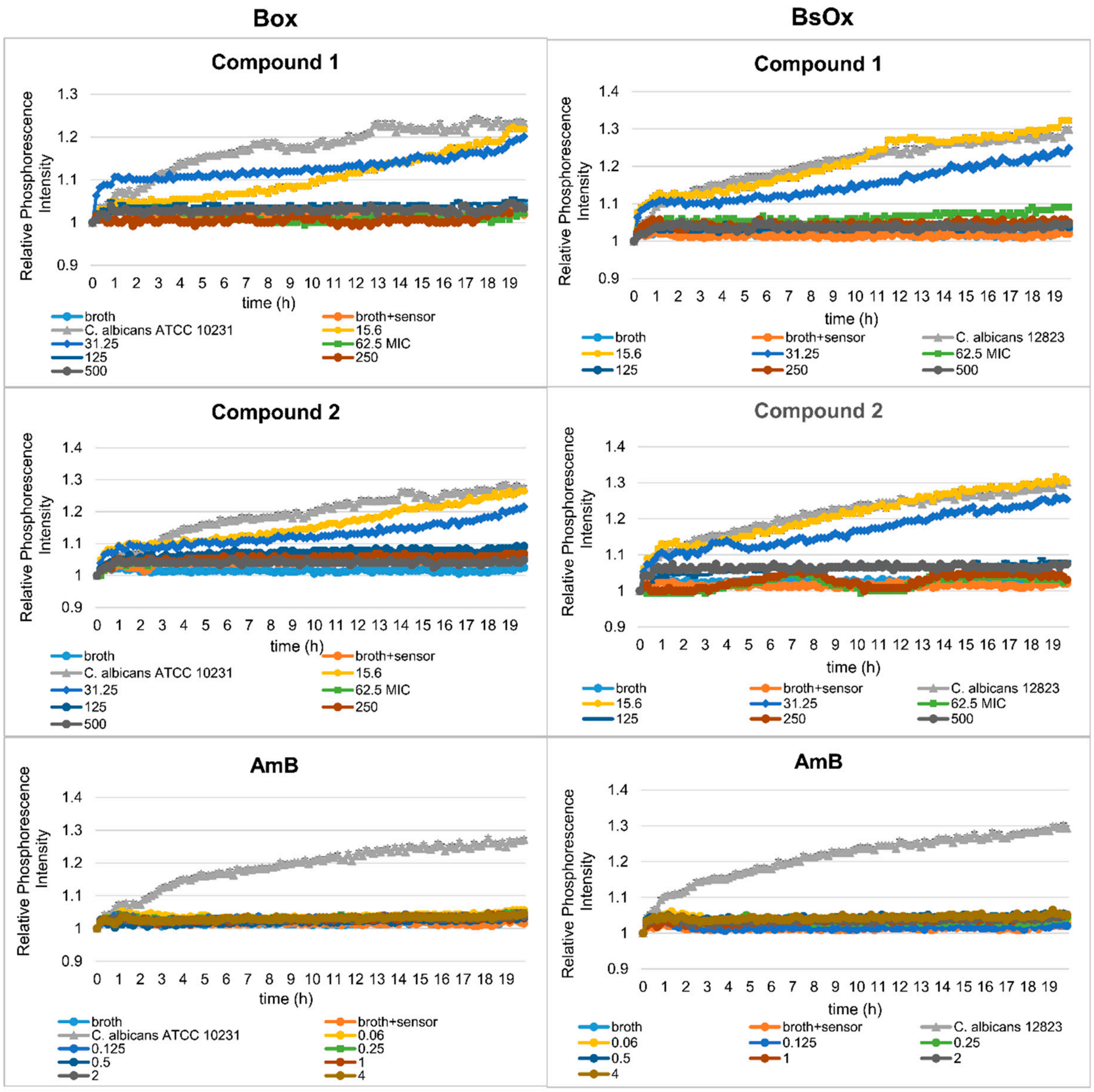

(a) *C. albicans* ATCC 10231 (b) *C. albicans* 12823

Figure S29. Profiles of relative phosphorescence intensity of **(a)** *C. albicans* ATCC 10231 and **(b)** *C. albicans* 12823 strains against different concentrations of compound (**1**), compound (**2**), and AmB with Box and BsOx (31.25 µg/mL) sensors. In the legend, the sample concentration is expressed in µg/mL.

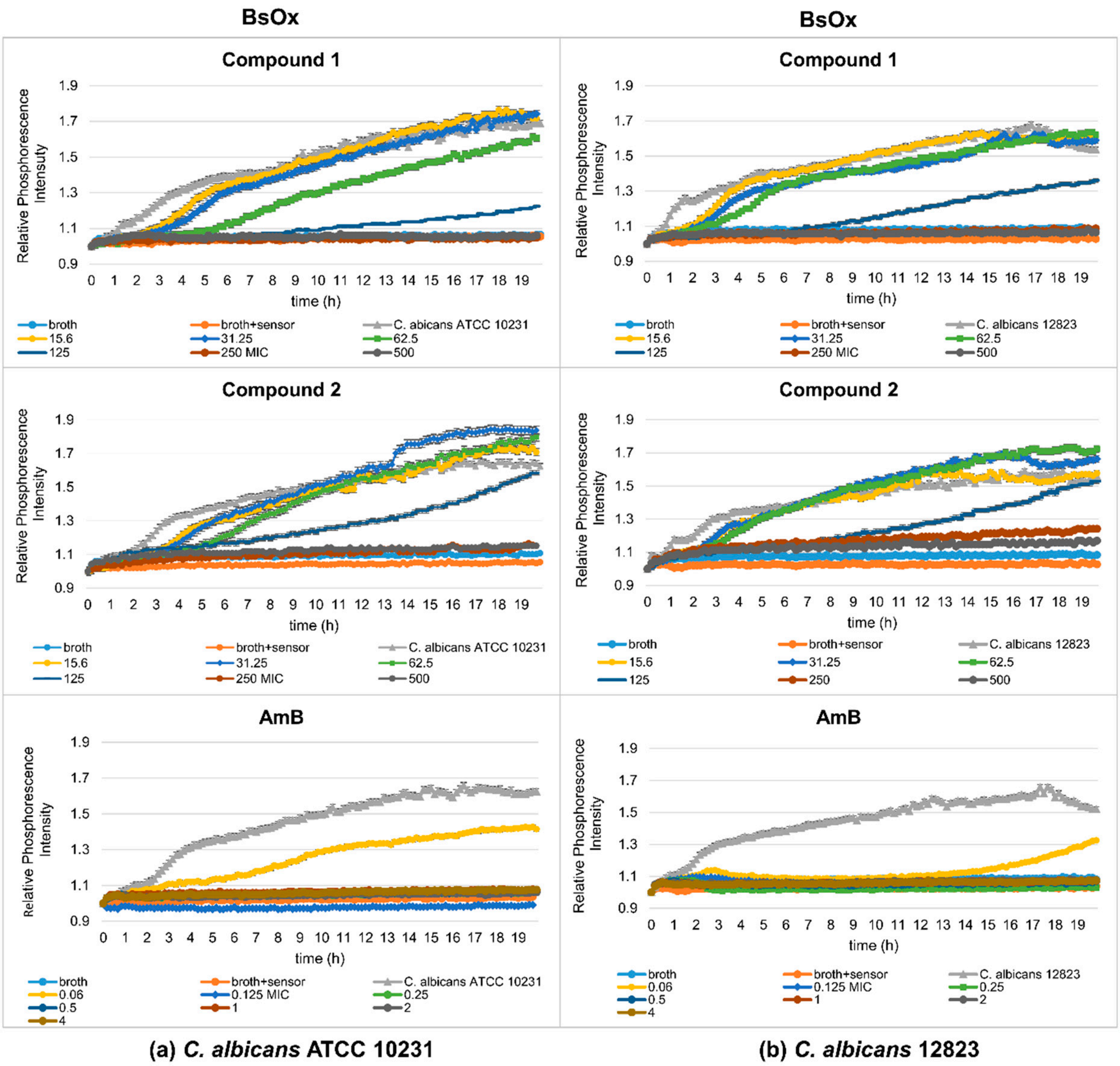

Figure S30. Profiles of relative phosphorescence intensities of **(a)** *C. albicans* ATCC 10231 and **(b)** *C. albicans* 12823 strains against different concentrations of compound (**1**), compound (**2**), and AmB with Box and BsOx (62.5 µg/mL) sensors. In the legend, the sample concentration is expressed in µg/mL.

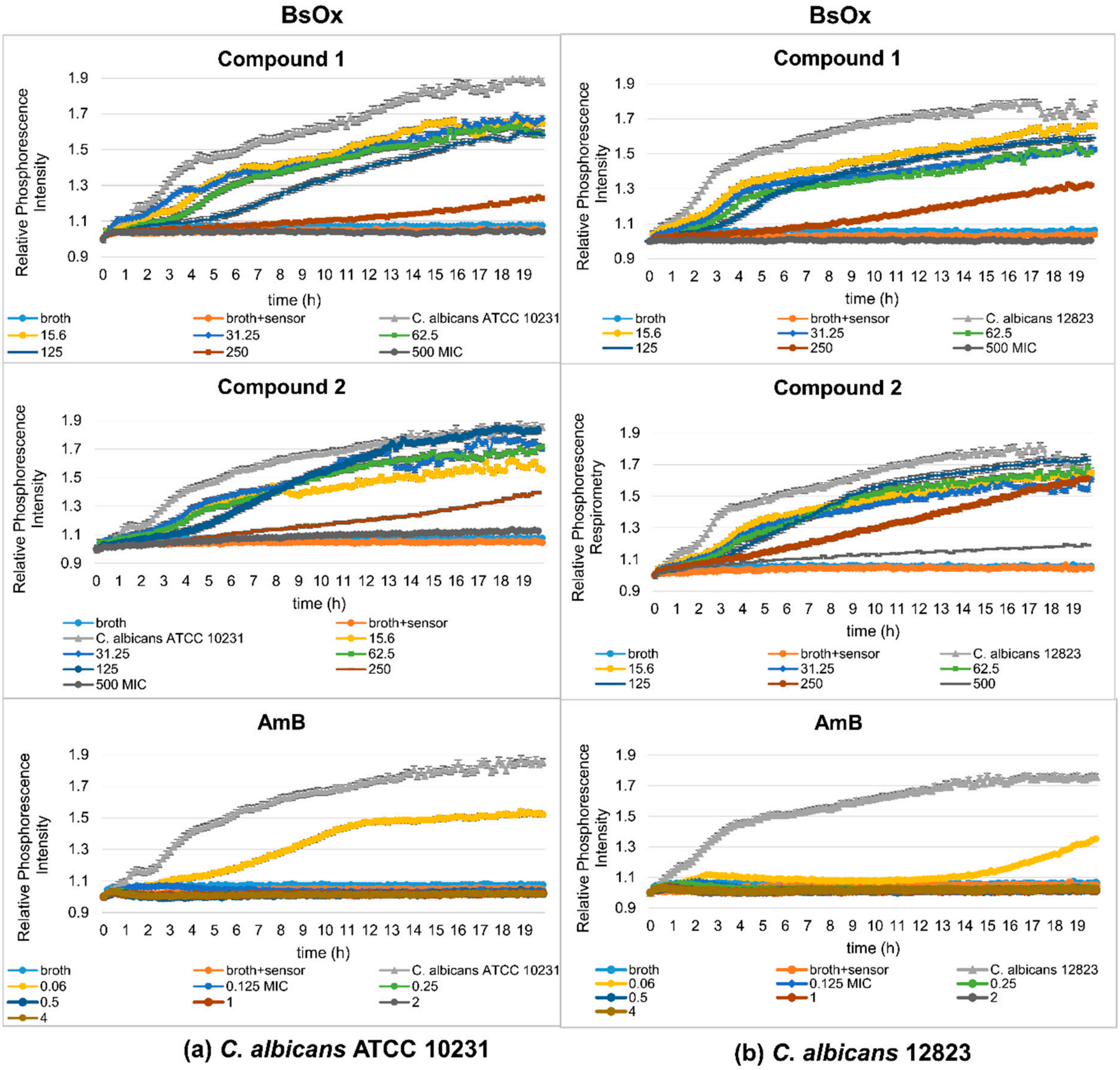

Figure S31. Profiles of relative phosphorescence intensities of **(a)** *C. albicans* ATCC 10231 and (b) *C. albicans* 12823 strains against the different concentrations of compound (**1**), compound (**2**), and AmB with Box and BsOx (125 μg/mL) sensors. In the legend, the sample concentration is expressed in μg/mL.
